# Mean human corneal diameter and palpebral fissure lengths as scales for forensic analysis of photographed faces: an analytical review*

**DOI:** 10.1007/s00414-026-03733-0

**Published:** 2026-02-23

**Authors:** Sean S. Healy, Carl N. Stephan

**Affiliations:** Laboratory for Human Craniofacial and Skeletal Identification (HuCS-ID Lab), School of Biomedical Sciences, The University of Queensland, Brisbane, 4072 Australia

**Keywords:** Eye, Iris, Anthropometry biometrics, Photograph, Focus distance, Average corneal diameter, Average palpebral fissure length

## Abstract

The quantitative measurement of facial photographs, termed photogrammetry, is an important procedure in multiple fields including ophthalmic, dental, bioanthropological, forensic, medical and other morphological sciences. When photographs are not life size, accurate measurements may be made by converting pixel lengths to millimeters, using a conversion factor, set by an object of known length, such as a line gauge, within the image. When an appropriate scale is not present, the conversion factor may be set by using an anatomical feature of known size. If the anatomical feature holds a narrow range of variation, a sample mean of the feature may, in some cases, suffice as a suitable substitute for an individual’s ground truth dimension. Due to their small variance, sample means of corneal diameter and palpebral fissure length have previously been used as scales, however, the vast published datasets have not been synthesized to triangulate on central tendencies. Herein we review methods and the full breadth of published data for adults and subadults (last five years) to calculate weighted pooled means and standard deviations for each anatomical structure. For the corneal diameter, these were 11.84 mm and 0.79 mm respectively (*n* = 296,887 eyes). The weighted pooled mean and standard for the most commonly measured 2D palpebral fissure dimension were 27.4 mm and 3.2 mm respectively (*n* = 5,871 eyes). A pilot trial of the pooled palpebral fissure length values for improved focus distance estimation in craniofacial superimposition using *PerspectiveX* is presented.

**Supplementary Information:**

The online version contains supplementary material available at 10.1007/s00414-026-03733-0.

## Introduction

There are many practical reasons why the examination of facial photographs is useful. For example: measurements may be more reliable when taken on static images than on the living person due to the absence of soft tissue compression; access to the living subject may no longer be possible (subject is deceased) and/or examinations may concern superimposition or overlay of image records that specifically are not possible from direct examinations of the subject alone. The conversion of quantitative measurements taken from facial photographs to life size is common when undertaking in these facial examinations for scientific purposes in anthropometry [1, 2], pediatrics [3], reconstructive surgery [4, 5] or the forensic sciences [6, 7]. For example, the derivation of life size measurements may be required or there may be a desire to compare with precision, two or more images that may be taken at different times. In the forensic science context this may concern 2D-2D image facial photograph comparison [8, 9], 3D-2D face superimpositions [10–13] and craniofacial superimpositions [14–16]—see Supplementary Material 1.

In the simplest context of a face photographed with both the scale and facial dimension of interest falling in the same plane and with the scale perpendicular to the line of sight of the camera [17–19], the procedure to calculate life size measurements is straightforward: divide the known metric length of the scale, visible in the image, by the pixel length recorded in the image to obtain a conversion factor. This factor can then be applied to any structures that lie within the same plane perpendicular to the line-of-sight of the camera, to calculate their physical ‘real-world’ value [19–22]. This procedure is well-known and has been used for many years, for example, in lateral cephalometry (X-ray images of the head and face) to help determine magnification factors [23–25]. Although accurate when performed correctly, errors associated with these photogrammetric techniques when performed incorrectly have been widely recognized and discussed [1, 19, 26–28].

Of course, when images are acquired for scientific or forensic purposes it is normal practice to include a suitable graduated line gauge within the image’s field of view to serve as the scale. However, images not acquired under such controlled settings may at times be the only ones available for scientific or forensic analysis. In these cases, some other object of known size that is recorded in the photographic image can serve as the scale to set a life size conversion factor [14]. A common example, when line gauges are not available is the use of coins since they are of known size [29–32]. Equally, anatomical structures of known size can serve as a reference object to set the scale when photographs concern the human body [33].

In facial photographs, when exact dimensions of face structures are not known, a sample mean of the structure can provide a good approximation of the individual’s anatomical trait dimension if the selected structure holds a tight range of human variation [20–22]. This of course only applies to a few limited facial structures with very small variance and where homogeneity is high across individuals typically of the same age and sex [20–22], but it nevertheless is an important attribute not to be discounted. This is not a novel practice, as anatomical features, such as corneal diameter, have been commonly used as scales in clinical settings for substantial time to enable measurements of faces as relevant to surgical interventions/outcomes [21, 34] and facial asymmetry [35, 36].

**Fig. 1:**
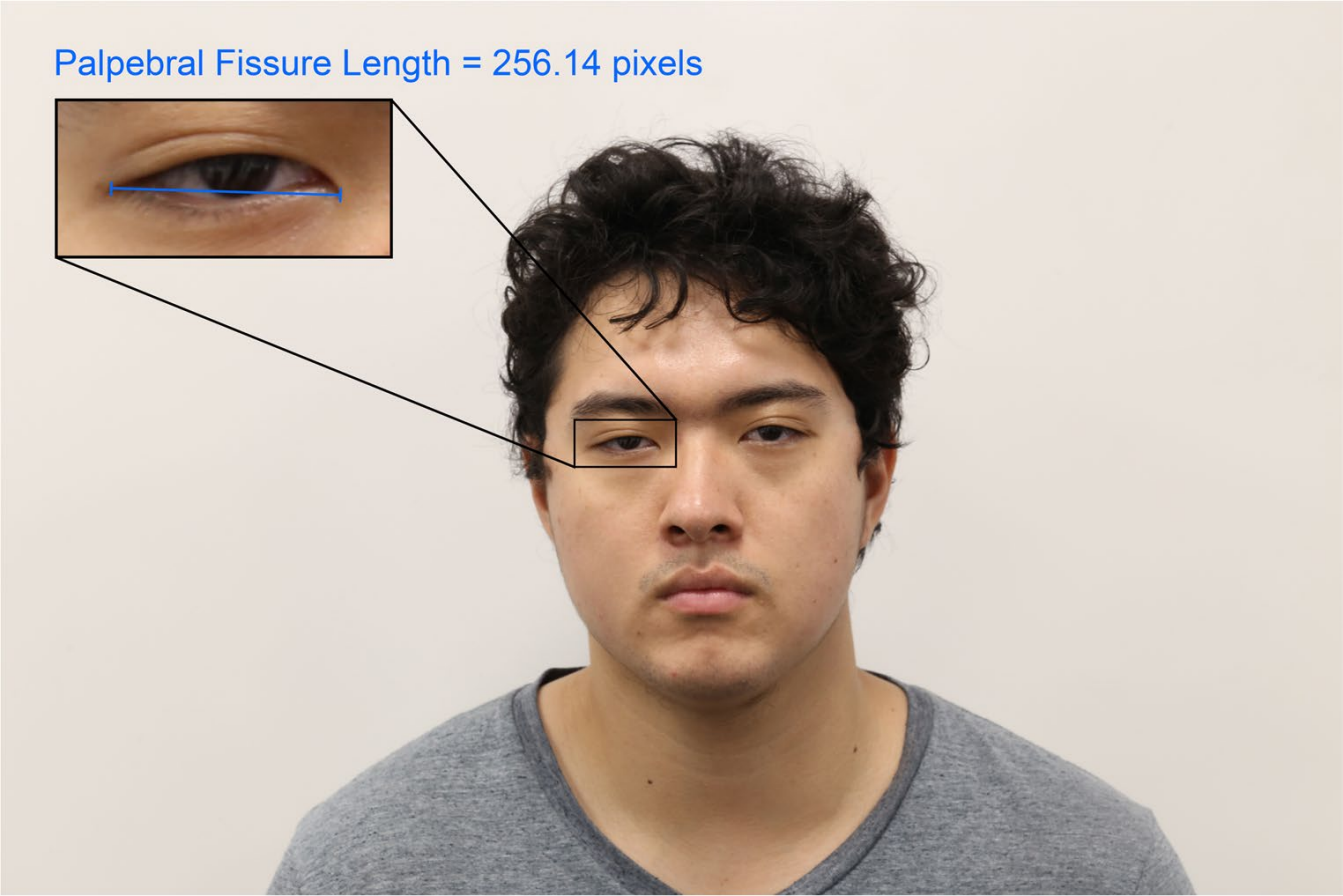
Example use of the palpebral fissure length, as a scale, to estimate the focus distance. For this image the ground truth focus distance was 2 m, and the image was taken using a Canon 6D camera body fitted with a 100 mm focal length prime lens. The estimated focus distance using *PerspectiveX* is 1.97 m per the following calculation (Eq. 1): Focus Distance = Focal Length × $\:\left(1+\frac{Sample\:Mean\:Palpebral\:Fissure\:Length\left[mm\right]\:}{Palpebral\:Fissure\:Length\:\left[pixels\right]\:\times\:\:Pixel\:Size\:}\right)\:$, Focus Distance = 100 × $\left(1+\frac{31.3}{256.14\;\times\;0.00655}\right)$ Focus Distance = 1.97m , [42]

In the field of forensics, anatomical scales within facial photographs serve another important and additional purpose relating to facial feature morphology. When the focal length of the lens used to take a photograph is known, an object of known dimensions can be used to estimate the focus distance—the distance between the camera and the object of interest—per the properties of similar triangles [37, 38]. Example focus distance estimation algorithms that follow this method include *PerspectiveX* [37, 39, 40] and MediaPipe Iris [41] (Fig. 1).

There exist two forensic techniques where these calculations are useful/important: Craniofacial superimposition and 2D/3D face superimposition (see Supplementary Material 1). Craniofacial Superimposition is a forensic technique where pre-existing reference facial photographs are compared to photographic images of a skull to determine whether the skull and face match for identification purposes [14–16]. This technique is often reserved for the most difficult to resolve cases involving skeletal remains, when the usual methods of identification through genetic analysis [43–46], dental radiographs/records [47, 48] and other medical radiographs [49–54], are not possible [37]. In this context craniofacial superimposition holds substantial practical significance because there are few other informative methods available to be used at that point. 2D/3D facial superimposition is similar and also requires focus distance calculations, however, it involves comparing a 3D face scan to pre-existing reference photographs [10–13]. This method also holds utility for identifying individuals from photographic images, such as those acquired from CCTV or traffic light cameras. The use of 3D scans allows for images taken from different orientation than a mugshot to be analyzed, as well as the comparison of point-to-point measurements within the two images [10].

Both of the above mentioned techniques rely upon the superimposition of a 2D representation of a 3D object onto a 2D reference image. For accurate comparisons to be made, the perspective of the two compared images must be the same. Specifically, the distance between the camera and the object of interest (face or skull) must match the distance between the camera and the face in the original reference photograph [18, 55–57]. Any mismatch of these distances (outside strict tolerance levels) will yield mismatching anatomy even if the objects are in fact derived from the same source subject (Supplementary Material 1). Subsequently, in 2D/3D Face superimposition and in craniofacial superimposition, it is critical that the focus distances for both sets of images are identical so that the image comparisons are valid [55, 56].

Up until 2017, the estimation of the focus distance from facial photographs lacking linear gauge scales was thought to be an impossible task within the craniofacial superimposition domain [42, 58, 59]. A recently introduced facial photograph focus distance estimation method using four input factors (the palpebral fissure length of the subject measured from the photograph in pixel units, the cameras pixel size in millimeters, a sample mean for the palpebral fissure length in millimeters and the focal length of the lens used to take the photograph) [37, 39, 40, 60] represents an important new contribution that breathes new scientific hope and legitimacy and life back into forensic craniofacial superimposition methods [37, 38, 55].

### Which anatomical features of the face are optimal scales and hold the narrowest variation span?

The best anatomical features to use as scales are those that are static or fixed, i.e., that do not move or change moment to moment in single individuals and/or are otherwise highly similar between different individuals. This rules out soft tissue structures that change shape with muscle contraction or facial expression, such as the mouth [61] or the pupil [62]. Traits with high potential include tooth crown dimensions, and certain structures related to the eyes, e.g., cornea size. While the crowns of the teeth hold small standard deviations (e.g., 0.39 mm for central canine width in males and 0.36 mm for females [63]), they are not always visible (reducing their utility). Unlike the teeth, the eyes are typically visible in facial photographs (photographs mid-blink are almost always retaken), and the eyes are often in precise focus and fall perpendicular to the camera’s line of sight, which is favorable.

The mathematical and physical properties of light travel and its functional constraints for functional biological apparatus also mean that the dimensions of various eye structures are tightly constrained [40]. Subsequently, the eyes represent good candidates to serve as linear gauges due to their narrow windows of anatomical variation. In particular, two anatomical structures of the eye are prime candidates: the corneal diameter, and palpebral fissure length. To date, the pre-existing published data on corneal diameter is extremely vast (hundreds of research datasets/papers) and that for palpebral fissure length is also sizable, yet these data have not been drawn together or synthesized in a comprehensive review. Here we provide an analytical synthesis of literature values for usage in facial photogrammetry and forensics and derived weighted mean and standard deviation values.

## Cornea

### Corneal dimensions

#### Background

**Fig. 2:**
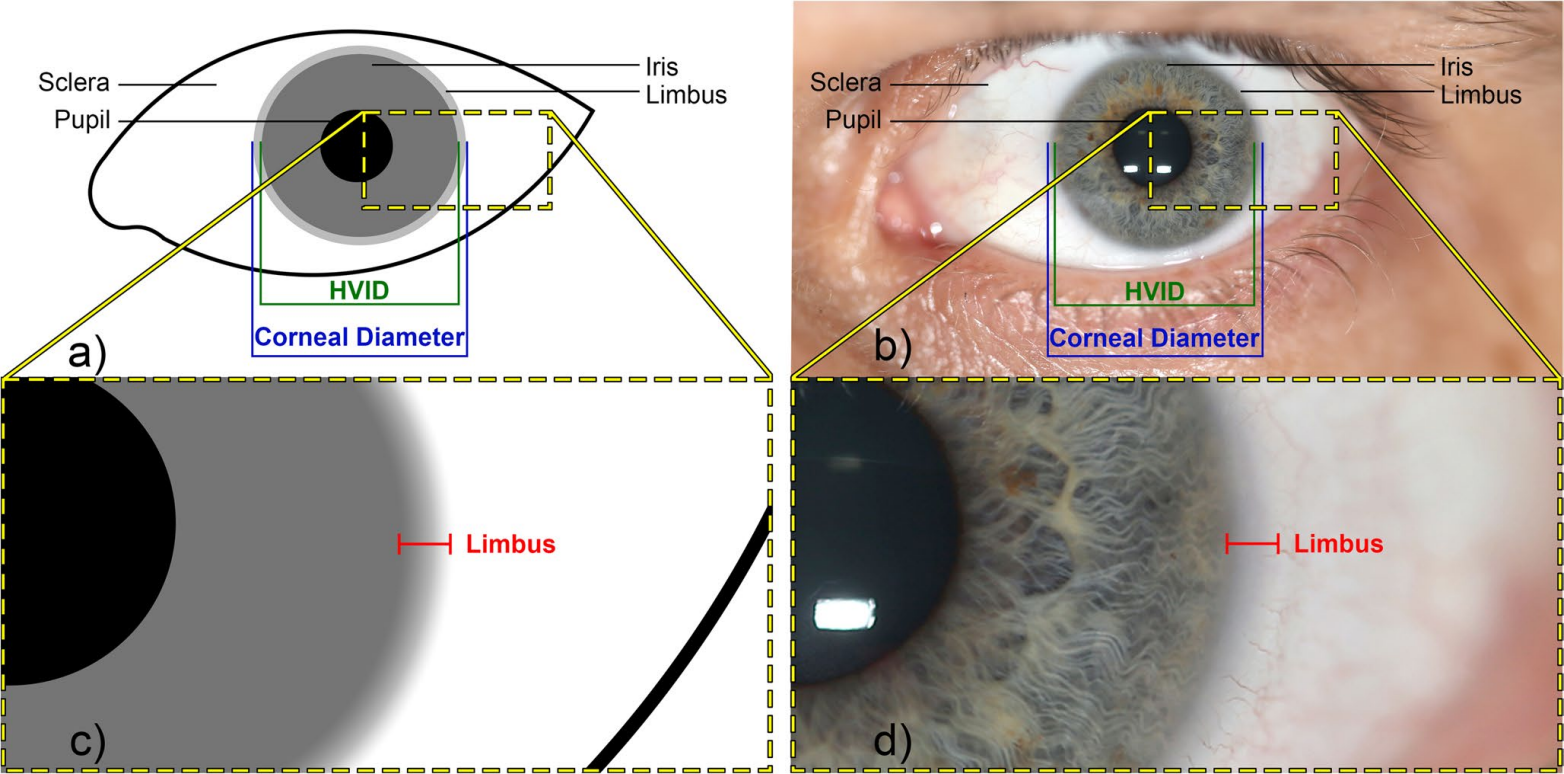
Corneal diameter and horizontal visible iris diameter (HVID): (**a**) diagrammatic representation; and (**b**) example on a real human subject. Dashed boxes show magnified regions

In anterior photographic views, the iris’ edge is relatively well-defined making it a small yet relatively distinct anatomical feature to measure. At the limbus, the iris gradually diminishes before merging with the white sclera posterior to the corneal junction. At magnified views, the iris’ edge is therefore not a clearly delimited structure with an abrupt transition, rather it represents a transitioning band [64]. This yields two similar, but different measurements of the iris/cornea commonly referred to as: (a) the horizontal visible iris diameter (HVID); and (b) the corneal diameter (Fig. 2). The horizontal visible iris diameter is the smaller of the two dimensions and represents a truncated measurement from the inner denser margin of the limbus, on either side, and measured horizontally across the iris [65, 66]. The corneal diameter, in contrast, differs by including the full thickness of the limbus, as measured from the outer less distinct boundary of the limbus at the junction with the white sclera and along the horizontal plane [67, 68]. It is worth pointing out that these two distinctly different measurements have sometimes been used interchangeably within the literature, which adds a degree of complexity/confusion [66, 69, 70]. Of course, if the corneal diameter is to be used as a metric scale, it is important to be clear about which one of the two measurements is being used. More information on the cornea anatomy can be found in Supplementary Material 2.

Complicating matters further, although the corneal diameter is almost exclusively used to refer to measurement on the horizontal axis, it should be noted that the corneal diameter can technically be measured across any axis of orientation [71], which is important because the cornea is not precisely circular. Rather, the cornea is wider than it is tall, making it an elliptical structure [71]. For example, Khng and Osher provide the metrics of 11.46 mm horizontally and 10.63 mm vertically [71]. In this paper, we exclusively reserve our attention only to the horizontal corneal diameter that includes the limbus as a contributing component to the measurement and representing the so-called ‘white-to-white’ measurement.

Corneal diameters are frequently recorded in the clinical and ophthalmology literature, both to diagnose pathologies, such as congenital glaucoma, but also for surgical planning, where the measurements are used to determine which replacement lens should be used [72–74]. These ophthalmology data carry dual utility for photogrammetry and forensic science, whereby corneal diameters can be used to set scale in images that do not include a gradated linear gauge as outlined above [22, 75–77].

Due to physical limitations of sampling, time and funding resources, the sample sizes for any given single study on corneal dimensions tends to be limited. For example, the commonly cited mean corneal diameter of 11.71 mm [22, 78–80] derives from a single study of only 390 healthy individuals [81], and excludes > 200,000 other data points that exist from other published studies. An aim of the present work is, therefore, to review all relevant recently published anatomical research data to provide pooled grand means derived from much larger sample sizes.

#### Measurements

As the corneal diameter literature is so vast (hundreds of studies, see e.g., Supplementary Resource 3), we only consider in this review, corneal diameters measurements recorded using optical biometers published in the last 5 years. Any studies concerning caliper measurements [82, 83] that typically hold larger measurement errors than optical biometers were excluded. Although a range of optical biometers utilizing different technology exist, the corneal diameter measurement is typically measured from a 2D image taken with a camera located inside the biometer [81, 84–87]. An algorithm is then employed to detect the start of the white sclera at its junction with the limbus, providing an automatic measurement of corneal diameter [68, 88]. As the optical biometer detects the junction border by the start of the white sclera on both sides of the eye (see e.g., Fig. 2), the term white-to-white (WTW) distance is commonly employed as a synonymous label for corneal diameter [74, 81, 84–87]. We include ophthalmology studies of both WTW distance and corneal diameter in this review.

Although the precision of optical biometers is submillimeter [89–92], the equivalency of data between instruments when measuring the exact same subjects is not always guaranteed [74]. Multiple studies have examined this aspect using the same subjects, revealing a range of (dis)agreements between instruments [74]. A review of the interchangeability of different optical biometers regarding white-to-white measurement reported a median difference between instruments of 0.65 mm, however, as much as 2.45 mm difference has been observed in some studies [74].

While comparisons can be undertaken between instruments, it is recognized within the ophthalmology literature that ultimately there is no definitive way to determine which instrument provides the best results or is the most accurate [74]. Human subjects do not come with accompanying ground truth values, and there is no consensus on a single instrument that serves as the gold standard [74]. In this context, calculating grand means across many studies using different biometers and calculating weighted results is useful since it provides a mechanism to triangulate upon underlying ground truth values by averaging out data noise resulting from various kinds of measurement error including that from instrumentation [93, 94].

#### Data review

A literature search was conducted in Scopus for publications published within the 5-year period between 2019 and 2024, which included corneal diameter means. Specifically, the search keywords used were: “corneal diameter OR white to white OR WTW”. Note that Scopus treats a space and a dash as equivalent, meaning that “white-to-white” was also included as a search term. Other criteria for the search included the medicine subject area and the English language. The literature search was conducted on 1-Aug-2024 and generated 1,044 articles. These articles were manually reviewed for relevance by the first author (SH). Papers were only retained if they reported: (a) a mean corneal diameter; (b) standard deviation of the corneal diameter; (c) the instrument used for measurement; (d) the number of individuals/eyes measured in the study. Additionally, all articles focusing only on neonates and/or infants (< 1 year of age) were excluded.

For articles where the same eyes were recorded across different experimental conditions, such as at different timepoints, only the data for the first reported session was retained. When studies included duplicate measurements of the same subjects, but recorded using different instruments, all measurements were included in this paper as there was no way to establish which instrument produced more accurate data (see Measurements above). Other exclusion criteria included: non-human animal studies, case studies, and pathologies that affect cornea size. In cases where different publications appeared to use data from some of the very same participants, only the article with the largest sample size was included in this review. For adults, a total of 234 articles in total were retained from the original 1,044 starting sample. A total of 14 subadult articles met inclusion criteria for this review.

For any instruments that were used in multiple studies, the weighted means and combined standard deviations were calculated using the equations in Supplementary Resource 4. Combined standard deviation was used under the assumption that the data were normally distributed, as has been specifically confirmed in some other studies [95, 96].

Where a single study used multiple instruments to measure the same eyes, data from only one instrument was used for the calculations of overall weighted mean and combined standard deviation. For adults, in these cases, which instrument was selected was based on its frequency of use within the 234 reviewed studies with preference being awarded to the most frequently reported instrument within the set. Additionally, the weighted mean and combined standard deviation were calculated by instrument to shed light on any long run instrument trends. As some studies provided incomplete instrument details or did not state which instrument model exactly had been used, some articles were pooled together in a miscellaneous category. This resulted in two generic Pentacam and IOLMaster groups, which span multiple different instrument models. Where name and model were provided for the Pentacam and IOLMaster biometers specifically, separate summary statistics for these instruments were calculated.

In cases where the same eyes were measured using multiple different instruments within the same single study, the weighted means, standard deviations and sample sizes of the subgroups by instrument were recorded for further analysis. In total, there were 77 different combinations of instrument pairs analyzed, where the exact same eyes were measured between at least two different instruments in the same study. This enabled mean differences, by instrument, to be specifically assessed (Supplementary Resource 5).

Due to the smaller number of subadult studies, only a single weighted mean and combined standard deviation was created from the grand total subadult dataset. Subadult data, for example, were not analyzed with regards to specific biometer instruments.

#### Adult results (subjects > 18 years)

The total adult sample included 296,887 corneas from 218,344 adults, measured using 38 different instruments (Table 1 & Supplementary Resource 3). Seven optical biometers were used to measure samples exceeding 5,000 corneas (which included left and right sides of some individuals per the original studies): IOLMaster 700, Lenstar LS900, Pentacam, Pentacam AXL, Pentacam HR and IOlMaster 500. Of these instruments, the largest corneal diameter weighted mean was recorded by the Lenstar LS900 at 12.03 mm (*n* = 30,282 eyes), and the smallest mean corneal diameters were recorded by the Pentacam instrument at 11.61 mm. (*n* = 14,667 eyes). On the whole, these values demonstrated negligible differences between measurement instruments. Measurement differences were larger for those instruments where fewer than 5,000 individuals had been measured, however, values were still within a 1.5 mm range. For example, the largest difference was observed between the Catalys femtosecond SD-OCT instrument and the Rev 80 instrument at 1.45 mm. The Catalys femtosecond SD-OCT recorded a weighted mean of 11.40 mm (*n* = 199 eyes), while the Revo 80 produced a mean of 12.85 mm (*n* = 144 eyes, see Table 1). The all-in grand tally produced a mean corneal diameter of 11.84 mm with a standard deviation of 0.79 mm (*n* = 296,887 corneas from 218,344 individuals, Table 1).

**Table 1:** Mean adult corneal diameter by instrument and ordered by pooled study sample size (number of eyes)

Instrument	Weighted mean (mm)	Combined SD (mm)	Number of eyes (*n*)	Number of studies (*n*)
IOLMaster 700	11.80	0.48	194,755	80
Lenstar LS900	12.03	0.51	30,282	32
Pentacam	11.58	0.41	14,667	15
IOLMaster	11.97	0.41	14,578	10
Pentacam AXL	11.61	0.39	13,058	16
Pentacam HR	11.74	0.51	9,270	24
IOLMaster 500	11.77	0.50	8,921	19
Anterion SS-OCT	11.88	0.49	2,803	22
Nidek OPD-Scan III	11.80	0.68	2,147	6
Tomey OA-2000	11.69	0.52	2,019	7
Orbscan 3	11.50	0.40	1,815	1
Argos SS-OCT	12.22	0.61	1,248	8
Nidek AL-Scan	11.88	0.42	1,072	7
Topcon Aladdin	11.54	0.46	889	5
Galilei G6	12.12	0.32	683	6
Tomey Casia 2 AS-OCT	11.62	0.61	667	7
EyeStar 900	12.06	0.42	645	4
Schwind Sirius	11.89	0.54	584	8
Visante AS-OCT	11.85	0.47	566	1
Orbscan II	11.65	0.40	557	6
Schwind MS-39	11.88	0.43	429	4
Quantel AB Scan Biometer	11.45	0.89	400	1
Suoer SW-9000	11.64	0.40	223	3
Catalys femtosecond SD-OCT	11.40	0.48	199	2
Colombo IOL 2	12.23	0.40	159	1
Alcon Verion	11.91	0.49	116	1
Revo 80	12.85	0.61	144	1
Galilei G4	12.15	0.40	142	1
Cirrus HD-OCT	12.02	0.51	122	2
ZW-30	11.95	0.42	82	1
Nidek ARK-1	12.01	0.44	65	1
Cassini Colour LED	12.65	0.52	49	1
Topcon MYAH	12.15	0.36	40	1
Scansys TA517	12.06	0.35	38	1
DRI-OCT Triton	12.40	0.48	33	1
Svision VG200	11.64	0.39	32	1
MR-6000	11.46	0.32	30	1
BQ 900	11.97	0.59	20	1
**Grand Results**	**11.84**	**0.79**	**296**,**887**	**234**

Instruments (that measured the same eyes) were often within 0.25 mm of one another. The two instruments with the highest agreement were the IOLMaster 700 and the Eyestar 900, with a mean difference of 0.01 mm (*n* = 458), whilst the largest difference was 1.03 mm, between the Revo 80 and the Pentacam AXL (*n* = 144) (Supplementary Resource 5).

#### Subadult results

**Fig. 3:**
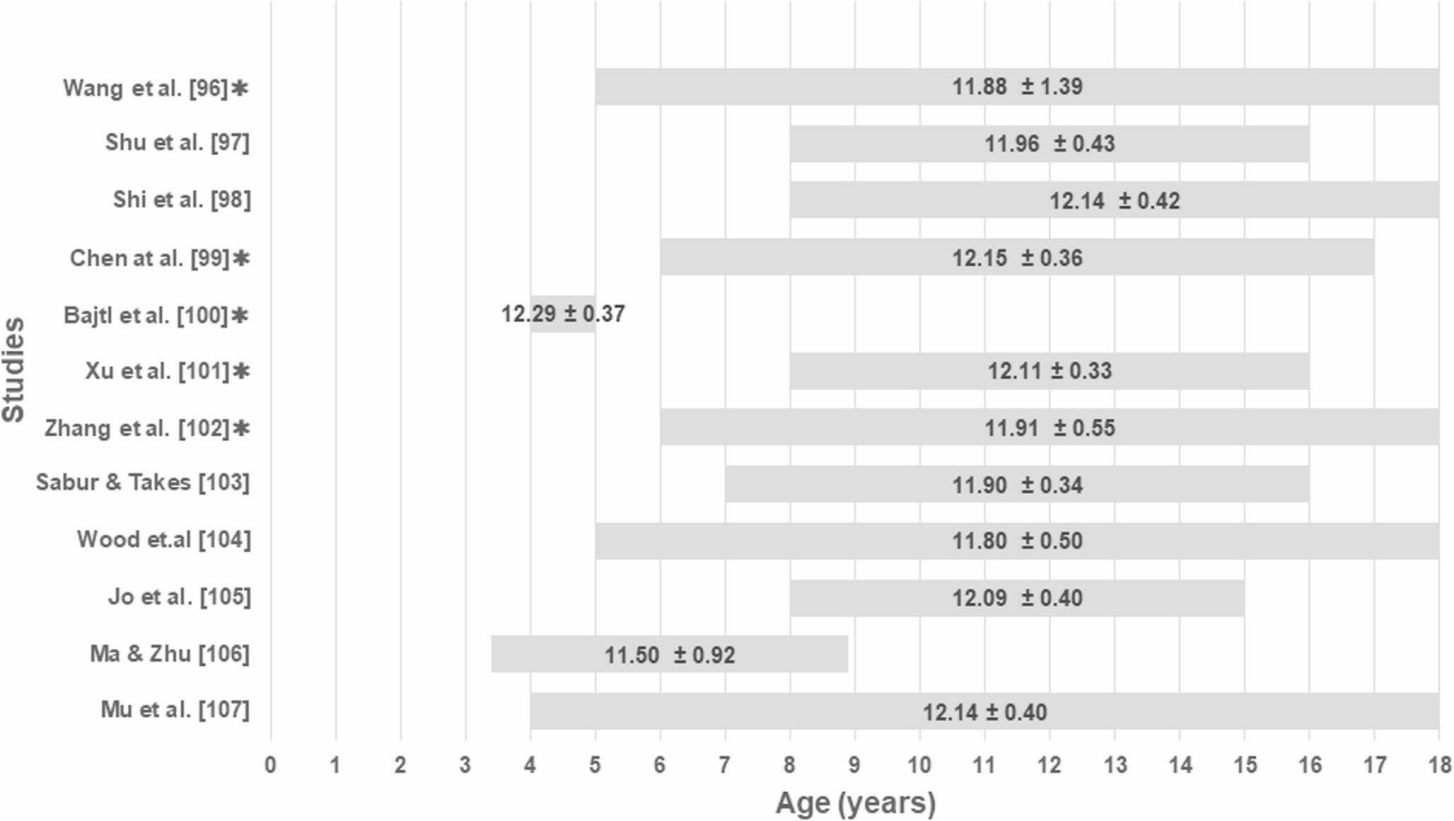
Subadult corneal diameter means, standard deviations and age ranges. ✱ Marks studies containing multiple sample subgroups, e.g., by sex, that were pooled here to report single summary statistics

The total subadult sample included measurements of 3,029 corneas of 1,817 individuals, aged between four and 18 years of age [97–108]. Note here that no studies conducted measurements by serially increasing subject age by single whole integer years, rather, investigators tended to study subjects within year bands that were not consistently defined between studies (Fig. 3). The overarching grand mean for subadult corneal diameter was 12.01 mm, with a weighted standard deviation of 0.60 mm. Per the data, the weighted mean results for subadults older than 4 years were essentially identical to values observed for adults. This is consistent with other observations that growth of the cornea largely occurs during the prenatal period and that adult corneal diameter is reached by one year of age [109].

## Palpebral fissure

### Horizontal palpebral fissure dimensions

#### Background

Horizontal palpebral fissure has also been suggested to provide a linear scale type calibration function due to slightly larger dimensions than corneal or iris diameter can provide and with commonly reported dimensions in the anthropometry and facial (trauma and plastic) surgery domains [110, 111]. The horizontal length of the palpebral fissure is, of course, defined by the metric distance between the endo- and exo-canthions, the two points where the upper and lower eyelids meet. Note here that the dimensional measurement may not run landmark-to-landmark through all three dimensions depending on the study, producing four different measurement varieties so far reported in the published literature (see Measurements below). More information on palpebral fissure anatomy can be found in Supplementary Material 6.

In subadults, the length of the palpebral fissure is clinically important and often used as a diagnostic marker for fetal alcohol syndrome in infants [112]. In the photogrammetry context, palpebral fissure lengths have been used to establish scale for focus distance estimation [37, 39, 40, 60] that, in turn, supports forensic examinations of the face and/or the skull [15, 18, 37, 38, 55, 113]. In the forensic context, this sees utility to the derivation of weighted means and combined standard deviations from pooled data as undertaken below.

#### Measurements

As mentioned above, four different types of palpebral fissure length measurements are clearly differentiated in the scientific literature (Table 2). Adding to confusion, across papers, palpebral fissure ‘length’ and ‘width’ are terms that are used interchangeably within and between studies giving a broadly ranging vocabulary. To lessen the chance of misinterpretation here, we reserve the term palpebral fissure *length* for the longitudinal measurement between the endo- and exocanthion through all three coordinate axes (x, y and z), while the label *width* is reserved for any horizontally orientated measurement that discounts one or more other coordinate axes when the longitudinal dimension of the fissure is measured along the x dimension (Table 2). Also, note that in this article, the x axis refers to the medial-lateral axis, y refers to the anterior-posterior axis, and z refers to the superior-lateral axis.

**Table 2:**
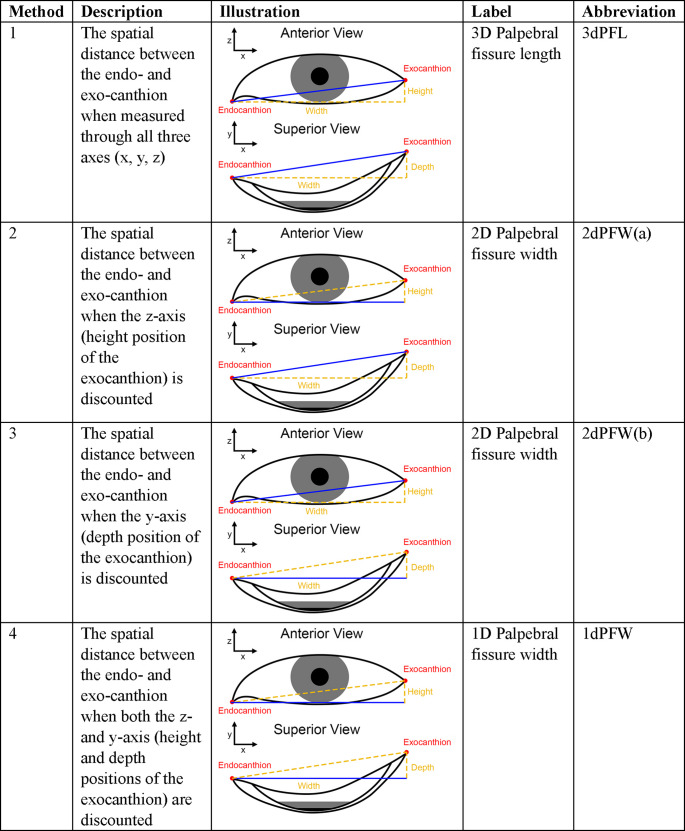
Four common methods of measuring the distance between the endo- and exo-canthion in the scientific literature. In each case, the corresponding length measurement is shown in blue

The most common palpebral fissure measurement reported in the literature for the past 5 years was 2dPFW(b), which represents a 2D width measurement discounting y-axis travel. This measurement, as well as 1dPFW, are measured exclusively from 2D photographic images, where it is impossible to measure depth along the y-axis in 3D space. In contrast 3dPFL and 2dPFW(a), which exclude no axes and the z-axis respectively, are typically measured directly on a living subject using instruments such as calipers and or digitizers [114–116]. Indirect measurement in this context is also possible, e.g., using 3D surface scans [117].

**Fig. 4:**
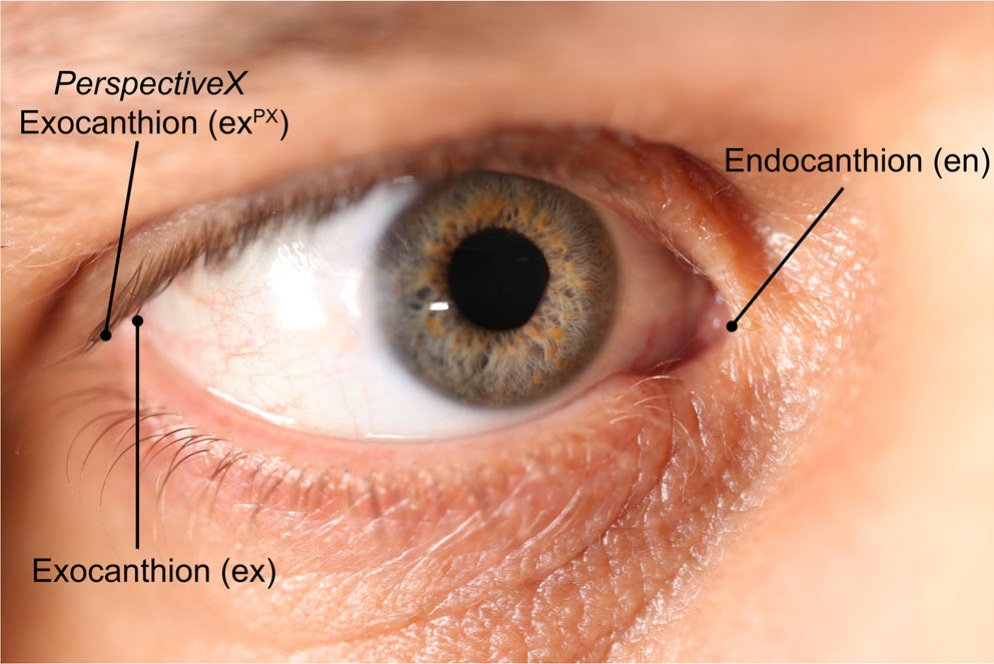
Oblique view photograph of eye showing the endo- and exocanthions together with the superior, posterior and lateral wrapping of the lower lid around the eyeball to its junction with the upper eye lid. The anatomical exocanthion is labelled ex. The exocanthion variation used by *PerspectiveX* is labeled ex^PX^

One additional definition of palpebral fissure length exists in the recent literature as created by Stephan specifically for *PerspectiveX* [37, 60].When measuring for *PerspectiveX*, exocanthion (ex) is placed on the external edge of the palpebral fissure lip, not on the scleral junction of inner edge of the eyelid (Fig. 4). For clarity, we label this landmark ex^PX^ to clearly differentiate it from ex, as the *PerspectiveX* variety. For the endocanthion, the traditional landmark is used, however, where endocanthion is obscured by an epicanthal fold, the meeting of the upper and lower eyelid at the epicanthal fold is used in *PerspectiveX* as a substitute for the endocanthion landmark [37, 60].

#### Data review

A literature search was conducted using Scopus and the keywords “palpebral fissure length OR palpebral fissure width”. Only articles in English were retained. The Scopus search was conducted on the 1-Nov-2024 and generated 298 articles. These articles were manually reviewed and sorted by the first author (SH) to retain only those that reported: (a) a mean palpebral fissure length; (b) standard deviation of the palpebral fissure length; (c) the instrument used for measurement; (d) the number of individuals/palpebral fissures measured in the study. Exclusion criteria included: animal studies, non-adult studies, case studies, pathologies that affect palpebral fissure morphology and any study that did not adequately define measurement instruments or techniques such that which coordinate axes contributed to the measurement were not clear or could not be unambiguously inferred.

In addition to the articles retrieved within Scopus, further articles were sourced through traditional methods of reference list searches. Note here that due to the relatively higher paucity of anatomical studies published on palpebral fissure lengths, this literature search was not limited to the last 5 years like that for corneal diameter. Palpebral fissure measurements were pooled by the four categories reported in Table 2 to calculate weighted grand means and combined standard deviations. Within each measurement category, sex specific subgroups were retained, in addition to calculating an overarching all-in grand total of combined sex samples.

#### Adult results

The total sample size of palpebral fissures measured for their length/width was 14,129 in 9,059 individuals, per the 38 total studies obtained (Table 3 and Supplementary Material 7). The means and standard deviations for 3dPFL, 2dPFW(a), 2dPFW(b) and 1dPFW were 32.6 ± 2.7 mm, 28.7 ± 4.7 mm, 27.4 ± 3.2 mm and 27.7 ± 3.7 mm respectively. A one-way ANOVA revealed that differences between all four different measurement classes were statistically significant (*p* < 0.05), clearly in part because of the large sample sizes.

**Table 3:** Mean dimensions for each one of the four palpebral fissure measurement classes (see Table 2) in adults

Measurement	Group	Weighted mean (mm)	Combined SD (mm)	Number of eyes (*n*)	Number of studies (*n*)
3dPFL	M	33.0	2.9	1,239	5
	F	32.0	2.3	1,117	5
	Combined Sexes (M + F)	32.5	2.7	2,356	5
2dPFW(a)	M	30.2	3.8	2,015	8
	F	28.9	3.6	2,277	9
	Combined Sexes (M + F)	29.5	3.7	4,548	11
2dPFW(b)	M	29.2	2.7	1,641	9
	F	28.1	2.8	2,387	10
	Combined Sexes (M + F)	27.4	3.2	5,871	14
1dPFW	M	28.6	2.7	463	6
	F	27.2	3.8	751	6
	Combined Sexes (M + F)	27.7	3.3	1,354	8

Analysis by sex (where studies reported sex separated data), generated male and female means and standard deviations for each measurement class. In general, males showed slightly larger (+ 1–3 mm) horizontal palpebral fissure dimensions than females, with the largest mean sex difference manifested for the 2dPFW(a) measurement, which discounted the y-axis translation. The male mean was 2.8 mm higher than the female mean in this instance.

#### Subadult results

**Fig. 5:**
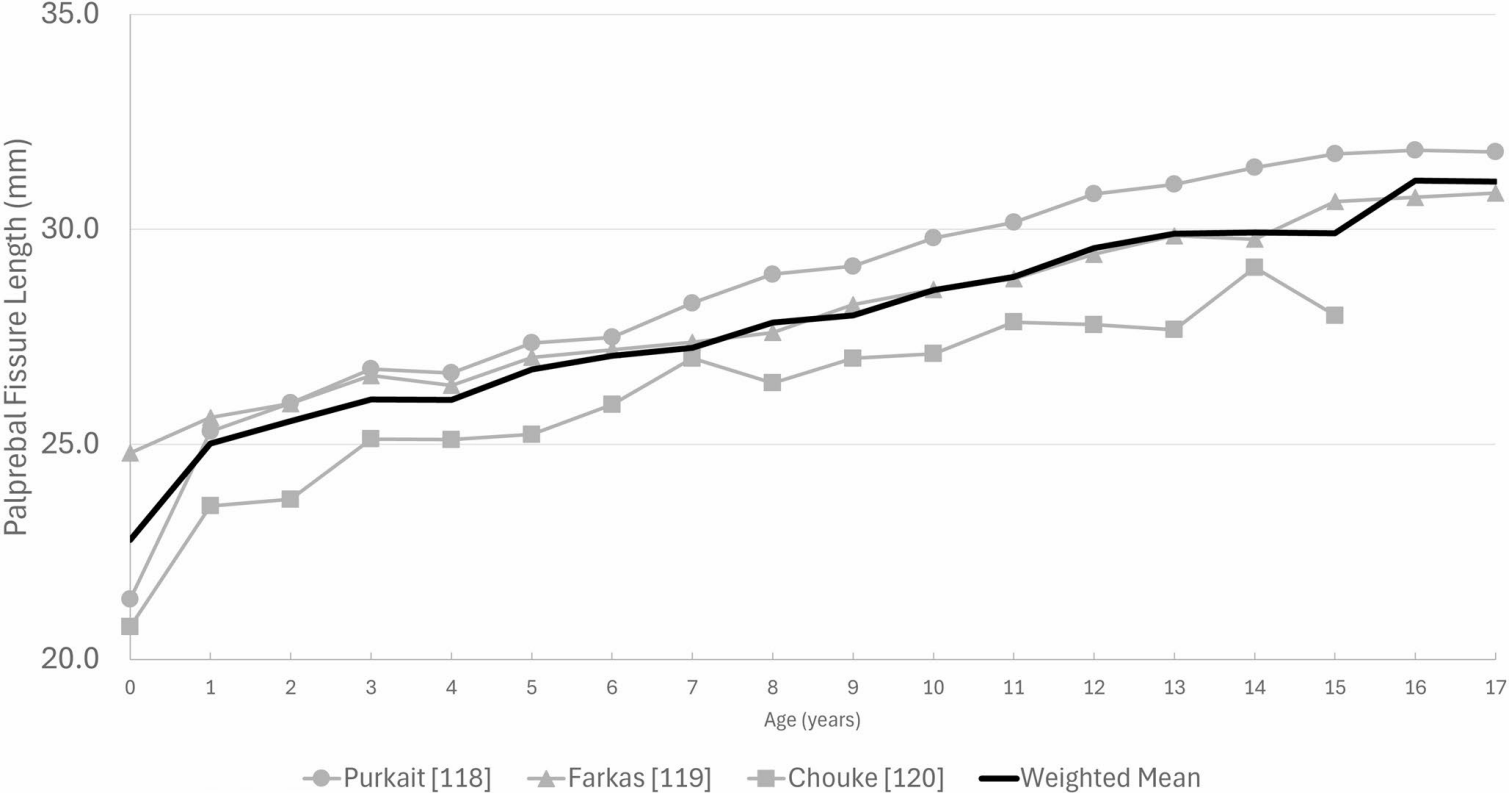
Subadult palpebral fissure lengths for studies reporting values at each whole integer age year and across the full 0–17 year age range of subadults. The weighted mean is shown in bold black font

Subadult palpebral fissure measurements were reported by prior investigators in two different styles, serially by single integer year ages, or grouped within multiple year age bands, the latter of which were not consistent between studies. Subadult corneal diameter means and standard deviations were calculated for each palpebral fissure length measurement, but were not split by sex due to sample size limitations (Table 4 and Supplementary Material 8). Figure 5 plots the data from studies which reported data by whole integer years [118–120]. These data indicate that around 1 year of age the palpebral fissure is approximately 77% of its adult size. This measurement increases in length fairly uniformly and consistently through to 16 years of age, where it has attained approximately 95% of the adult value.

**Table 4:** Mean dimensions for each one of the four palpebral fissure measurement classes (see Table 2 ) in subadults

Measurement	Weighted mean (mm)	Combined SD (mm)	Number of eyes (*n*)	Number of studies (*n*)
3dPFL	29.4	3.1	4,258	4
2dPFW(a)	25.9	1.0	3,510	3
2dPFW(b)	28.6	3.3	1,844	1
1dPFW	26.9	2.7	524	3

### Real world pilot tests of newly derived palpebral fissure length grand means as standards for *PerspectiveX*

 Previously, Farkas data [119] has been recommended for use with *PerspectiveX* to estimate focus distances [37, 60], however, with increased sample sizes for the grand means reported here, it is useful to consider if these new means offer improved benefits to focus distance estimation. Subsequently, we used five photographs each belonging to each of the authors (10 photographs total) at known focus distances between 2 and 6 m and all combinations of the 10 reference means (two from Farkas [119] and eight new pooled means from this study) and 3 different ways of measuring the palpebral fissure length on the facial photograph (en-ex^PX^ or en-ex; see Table 5) to provide preliminary insights on what data combinations work best.

**Table 5:** Absolute mean error (m) of focus distance estimations made using *PerspectiveX*. The bolded values indicate the lowest errors in each data column

Input Reference Mean for *PerspectiveX*		Type of Palpebral Fissure Length Measurement from the Facial Photograph for *PerspectiveX* analysis		
		Palpebral Fissure Length per Stephan’s [18] original en-ex^PX^ formulation	en-ex after 2dPFW(b) (Method 3, Table 2)	en-ex after 1dPFW (Method 4, Table 2)
Male	Farkas [119]	0.23	0.53	0.54
	3dPFL Grand Mean [this study]	0.43	0.70	0.71
	2dPFW(a) Grand Mean [this study]	**0.18**	0.41	0.42
	2dPFW(b) Grand Mean [this study]	**0.17**	0.31	0.32
	1dPFW Grand Mean [this study]	**0.17**	0.25	0.25
Combined	Farkas [119] Combined Mean	0.22	0.49	0.49
	3dPFL Grand Mean [this study]	0.37	0.66	0.66
	2dPFW(a) Grand Mean [this study]	**0.17**	0.34	0.35
	2dPFW(b) Grand Mean [this study]	0.31	**0.20**	**0.18**
	1dPFW Grand Mean [this study]	0.27	**0.20**	**0.19**

The lowest mean error (0.17 m) was observed in three instances, all using the en-ex^PX^ measurement from the facial photograph, and in combination with the reference means of male only 2dPFW(b), male only 1dPFW and combined sex 2dPFW(a). The highest mean error (0.71 m) was observed when using the 3dPFL grand mean of this study, as the real-life palpebral fissure length in combination with the 1dPFW class of measurement from the 2D facial photograph.

## Discussion

### Statistical benefits of pooling data from multiple studies

The pooling of data undertaken here to calculate weighted grand means and combined standard deviations is useful to maximize statistical reliability under the Law of Large numbers. In other words, statistical noise belonging to any particular study will tend to be averaged out across the full suite of studies to provide more reliable statistics that are based on much larger sample sizes. In this manner, the pooled data provides a triangulation to underlying population parameters that would otherwise remain elusive in the single study context where limitations of representativeness, possible systematic errors of single instruments, and small sample sizes are all typical considerations [93].

Data pooling additionally holds value in circumstances where multiple different instruments have been used to measure the same anatomical feature, all producing slightly different results, and without consensus as to which one is preferred/most valid [74]—this is most strongly applicable to the corneal diameter data. Data pooling not only removes random noise but will additionally average out systematic errors of different instruments when they are in opposite directions around the underlying ground truth. In this review, this approach of data pooling enabled overarching summary statistics for corneal diameter to be derived from 234 studies, using 38 different optical biometers, and from a total sample size of 218,344 surveyed individuals, as published in the last 5 years.

### Glimmers of residual sampling and measurement errors in the pooled dataset

A comparison of the grand means from pooled adult and subadult corneal diameter datasets suggests that some sampling effects and measurement errors continue to persist in the pooled data and that the protocols described above were not entirely successful in eliminating every trace of data noise. These data discrepancies are useful to acknowledge as they provide an indicator as to the reliability and precision of the (current) pooled sample data. For example, it is evident that the adult grand mean for corneal diameter is slightly less (11.84 mm, *n* = 218,344) than the 4–18 year-old subadult grand mean (12.01 mm, *n* = 1,817). As the corneal diameter is unlikely to be shrinking in adults (adult size is attained at 1 year of age and remains consistent thereafter [121]), this 0.17 mm difference thereby likely represents ongoing residual noise due to the inexact human enterprise of scientific data collection in cross-sectional samples.

It is reasonable to speculate that this small persistent noise is more likely to be a problem for the subadult data given its the much smaller sample size (3,029 corneas of 1,817 individuals) in contrast to that for the adult cohort (296,887 corneas from 218,344 individuals), but until further data are collected to permit re-evaluation of this attribute, the nature and source of this small oddity can only be theorized. As the difference is very small (0.17 mm), and less than outer margin of instrument error designated to be clinically meaningful (0.5 mm [74]) the difference can, in the applied practical sense be entirely disregarded. That is, the subadult (4–18 yrs) and adult values (18 + yrs) can be considered equivalent. Also note here that the subadult and adult age brackets do not include the same individuals, i.e., they are independent, which is likely to be another source of ongoing data noise.

For palpebral fissure width, evidence of the ongoing persistence of noise and independent sampling effects in the pooled data are larger, which is not entirely surprising since the pooled sample sizes for this anatomical structure are smaller than those for corneal diameter. For example, the 2dPFW(b) grand mean (27.4 mm) was one millimeter smaller than the 1dPFW measurement (28.4 mm). All else being equal, these differences are in the reverse direction to that expected via trigonometric relationships, where longer distances are expected when the same measurements are undertaken through more cartesian axes. Thereby, statistical wobble in the weighted palpebral fissure statistics continues to be present despite the advantages of data pooling and reaches the magnitude of a whole millimeter units. Care should, subsequently, be exercised not to over interpret the precision of the pooled palpebral fissure length/width data based at submillimeter levels of precision.

### Strength and limitations of each anatomical trait as a metric scale for photogrammetry

In terms of their utility of these anatomical structures as metric scales in photogrammetry, two things are critical: (1) it is important that a mean value for the anatomical trait is closely representative of a very large percentage if not all individuals; and (2) that the anatomical structure can be accurately measured on the reference object with little error. We discuss these items separately below.

#### Corneal diameter

Corneal diameter holds the advantage that the anatomical structure has been commonly measured in a consistent way across many studies, such that the pooled data are derived from a very large sample size (e.g., *n* = 218,344 adults) essentially assuring high reliability of the summary statistics. The anatomical structure also holds a narrow range of variation (combined standard deviation of 0.79 mm in adults), such that the central tendency statistic of 11.84 mm (arithmetic mean) is a good approximation for many individuals within the distribution. That is, 95% of individuals who fall between − 2 and + 2 z-scores, will not differ to the pooled mean by more than 1.6 mm as a maximum absolute value. One standard deviation (0.79 mm) also represents a small percent size (6.7%) of the pooled mean, such that the sample data are clustered in a tight cloud and well represented by the arithmetic mean as the central tendency statistic.

A limitation to the corneal diameter as an object of known dimension is its overall small size, with a mean of only 11.84 mm, such that very precise measurement in a face photograph is desirable, since any measurement error will be large in relative percentage terms to the structure’s overall small size. This limitation is offset somewhat by the relatively abrupt change from iris to the sclera at the limbus, but even so, the area of this transition is a gradual zone rather than a precise line (Fig. 2) that adds challenges to manual corneal diameter measurement from face photograph, especially ones with low resolutions. Such non-ideal lower resolution images ahold relevance for forensic image analysis since they are commonly occurrences. A final limitation, which also applies to palpebral fissure length, is that these features can only be used when visible within a photograph. Whilst face photographs will typically include open eyes in clear view, headwear such as sunglasses may obscure these features.

A factor not considered within this study was age-related changes of the cornea. Data were collected from a large age range of adults (18–100 years old), and as such the generated weighted mean represents adults of all ages. Previous studies have reported statistically significant negative correlations between age and corneal diameter; however these correlations were weak (*R* = −0.21 [122] and *R* = −0.16 [81]). It has been suggested that the changes in corneal diameter measurement may not be a true change in length, but due to pathologies such as arcus senilis, which may cause inaccuracies in automatic measurement. Bracketed age means therefore may have lower variance than the overall weighted mean and could be explored in the future.

For corneal diameters, it is relevant to note that the subadult (4–18 yrs) data that were drawn from the literature were less variable than those of adults, i.e., subadults held a combined standard deviation of 0.60 mm or 5% of the central tendency dimension. We hypothesize that this tighter subadult data range may be due to a smaller breadth of measurement instruments being used for the subadult data cohort (four) compared to the adult dataset (38 instruments).

#### Palpebral fissure length/Width (PFL/W)

2dPFW(b) held the largest sample size for PFL/W at *n* = 5,871 individuals, which is sizable and adds reliability compared to any smaller sampled single study of palpebral fissure width in isolation. The range of variation for this measurement was also small (3.2 mm) relative to the mean dimension of the trait (27.4 mm), but the percent size was approximately twice that of the corneal diameter (11.7 versus 6.7%). Subsequently, the PFL/W data were not clustered as tightly as the corneal diameters. On the other hand, an advantage of the PFL/W as a scale is its larger mean dimension—almost three times that of the corneal diameter—such that pixel unit error within measurement has a smaller impact on the scale factor and thus real-life measurement conversion compared to corneal diameter.

**Fig. 6:**
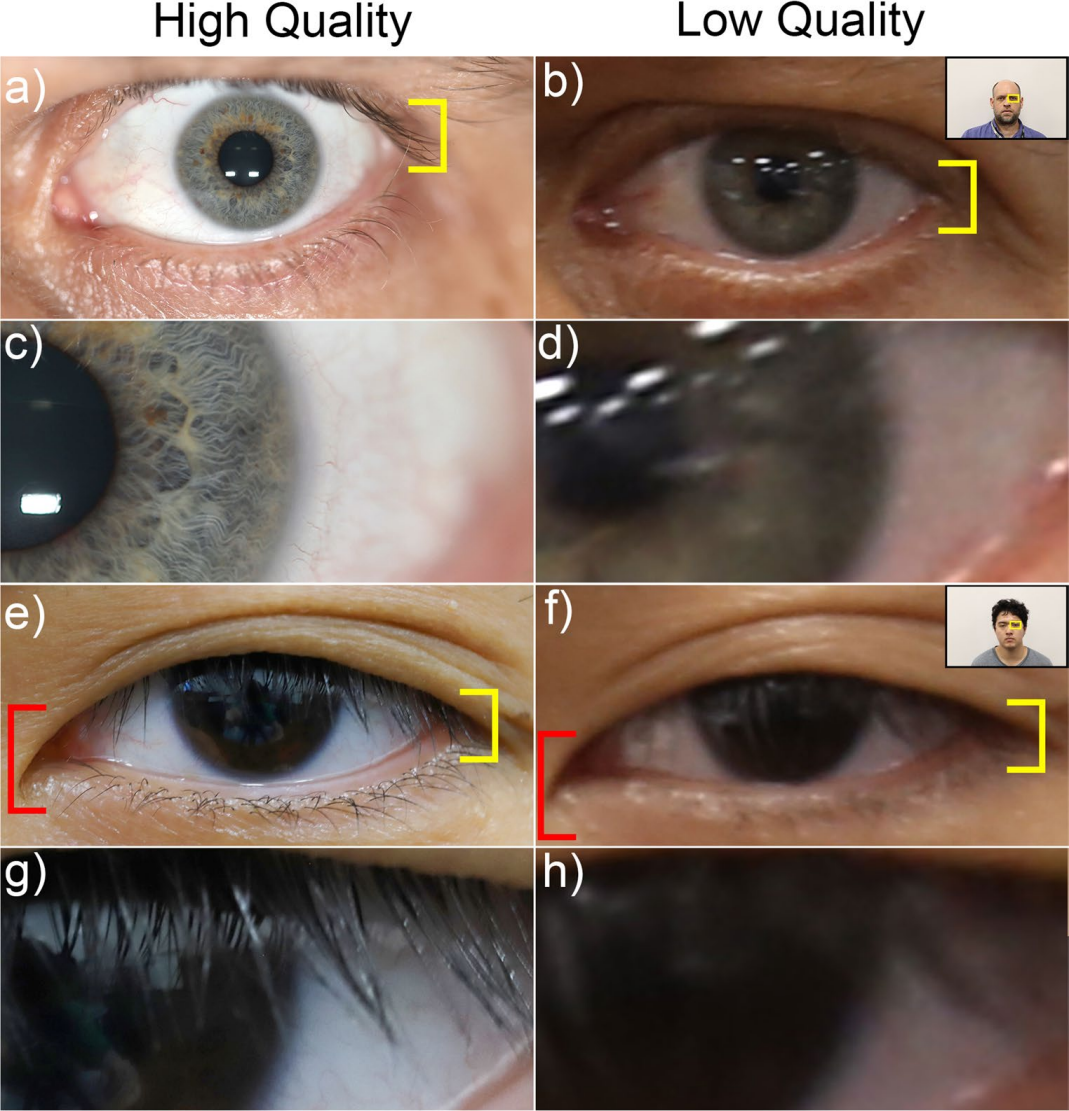
Challenges in defining corneal diameter and palpebral fissure width from images of differing resolution quality: (**a**) CS’s left eye (last author) where the precise position of the exocanthion landmark is obscured by the upper eye lashes (yellow bracket). (**b**) a lower resolution version of the same feature in a), derived from a larger field of view image (see embedded thumbnail). (**c**) a magnified view of the limbus from (**a**) (high resolution view of the eye) (**d**) a magnified view of the limbus from (**b**) (low resolution view of the eye). Note the blurring of the limbal region and the prominence of its inner rather than outer margin. (**e**) SH’s left eye (first author) where the precise position of the exocanthion landmark is obscured by the upper eye lid and its lashes (yellow bracket) represents a low-quality face photograph image of Author 2. f) a lower resolution version of the same feature in e), derived from a larger field of view image (see embedded thumbnail) represents the same image in a close-up view of the limbus. (**g**) a magnified view of the limbus from (**e**) (high resolution view of the eye) (**h**) a magnified view of the limbus from (**f**) (low resolution view of the eye). Note the blurring of the limbal region and the prominence of its inner rather than outer margin

A downside to PFL/W as a scale for photogrammetry, is that the exocanthion may be obscured by eyelashes [37, 40, 60], some epicanthal folds [123, 124] or ptosis [125–127] (see e.g., Fig. 6), making traditional exocanthion and the palpebral fissure dimension difficult to unambiguously establish.

Four palpebral fissure dimensions exist, such that which one to use as a scale for life-size adjustment of 2D photographs is an important consideration. As 2D face photographs only permit 2D measurements, 2D palpebral fissure widths from Table 2 are arguably the most directly applicable. Furthermore, 2dPFW(b) appears to be the most appropriate dimension because it is a point-to-point measurement that maximizes the number of coordinate axes for the length measurement in the 2D context.

Similarly to corneal diameter, age-related changes of the adult palpebral fissure were not considered with adult data pooled within each palpebral fissure definition. Both endocanthion and exocanthion have been reported to change their position on the face with age [128], however the changes to palpebral fissure length are not clear. Multiple studies have examined the effect of age and have provided a variety of results. While several studies reported upwards of three mm decrease in length in their oldest age groups compared to their youngest, other studies reported changes under one mm or even increases [115, 116, 129–133]. Further research to determine the effects of aging should be conducted in the future.

Generally, the breadth of palpebral fissure data that exists in the published literature is under recognized in the applied context with only a few hallmark studies, such as Farkas et al. being well-known or widely cited. This is one of the drivers for undertaking the palpebral fissure data reviews in this study. For instance, the *PerspectiveX* algorithm [37, 39, 40] has previously been engaged with the 3dPFL data of Farkas [119], but *PerspectiveX* concerns 2D facial photographs. Subsequently, while *PerspectiveX* has been extensively cross-validated and shown to work on the Farkas et al. data [37, 39, 40], there nevertheless exists a mismatch between its 2D applied photographic context and the 3dPFL reference data upon which it currently engages. The potential thereby exists to optimize and improve *PerspectiveX* further by more refined and strategic selection of the palpebral fissure reference data specific to the 2D photographic context at hand. This is one of the drivers to undertake the current published reference data review in this study.

####  Utility of the pooled palpebral fissure means for *PerspectiveX *(and a comparison to Farkas’ 3dPFL means [119])

 For focus distance estimation using *PerspectiveX*, Farkas et al.’s data [119] have previously been recommended as a matter of convention [37], as mentioned above. It is subsequently useful to assess how all four of the new pooled palpebral fissure dimensions derived in this study perform with *PerspectiveX*, which ones a provide the best focus distance estimation results for forensic casework, and whether or not the pooled means outperform Farkas et al.’s means.

The pilot trial of this study using 10 photographs of the two authors, suggests that when a sex specific (male) pooled means of 2dPFW(b) and 1dPFW provide the best focus distance estimation results and when the palpebral fissure was measured according to the *PerspectiveX* definition (en-ex^PX^). These pooled means also outperformed *PerspectiveX* when utilized with the Farkas data, i.e., estimation error was reduced by a mean of 0.06 m overall for all 10 photographs, Table 5. It is notable, however, that the results did not remain consistent for the sex combined grand means. Rather, in this context 2dPFW(a) provided the best results, again using the *PerspectiveX* definition for palpebral fissure width (en-ex^PX^). The error reduction in this circumstance, in contrast to the Farkas 3D reference data, were again − 0.06 m, Table 5. While these results suggest that the pooled data offer improved value over and above the Farkas reference means, the inconsistency between sex specific and combined data suggest that further validation testing in larger samples of subjects and photographs would be beneficial.

It is interesting to note that when the *PerspectiveX* methods for measuring the palpebral fissure length (en–ex^PX^) on the facial photograph is substituted for the combined sex 2dPFW(b) and 1dPFW measurements using en-ex, the focus distance estimation errors were also very small. This suggests that combined use of 2D facial photograph measurements of palpebral fissure length with 2D reference data may offer further enhancements. Again, further larger scale validation tests using larger numbers of subjects and images will be useful to verify the legitimacy of the pilot results observed here to confirm utility in/for forensic settings.

## Conclusions

Data representing 297 studies and 324,271 measurements were used to generate weighted pooled means for corneal diameter and four separate definitions of palpebral fissure length for both adults and subadults. Combined standard deviations were calculated for each mean, showing that the data tightly clustered around the means, allowing them to serve as accurate scales within photographs. Finally, a pilot study comparing the generated means with previously used Farkas values for focus distance estimation showed the pooled values offer an improvement, but further validation studies should be conducted in the future.

## Supplementary Information

Below is the link to the electronic supplementary material.

Supplementary Material 1

Supplementary Material 2

Supplementary Material 3

Supplementary Material 4

Supplementary Material 5

Supplementary Material 6

Supplementary Material 7

Supplementary Material 8

## Data Availability

All study-specific data used for corneal diameter and palpebral fissure contained herein are available in the scientific domain and can be readily accessed from there. For corresponding referenced papers/works see citations provided in Supplementary Material 5, 7 and 8.
