# Supplementary material for: Mean human corneal diameter and palpebral fissure lengths as scales for forensic analysis of photographed faces: an analytical review*
Source: Int J Legal Med. 2026 Feb 23;140(3):1529–46. doi: 10.1007/s00414-026-03733-0 (PMC13161299; doi:10.1007/s00414-026-03733-0)
Supplement: Supplementary file 1 — Supplementary Material 1 [file 414_2026_3733_MOESM1_ESM.docx]

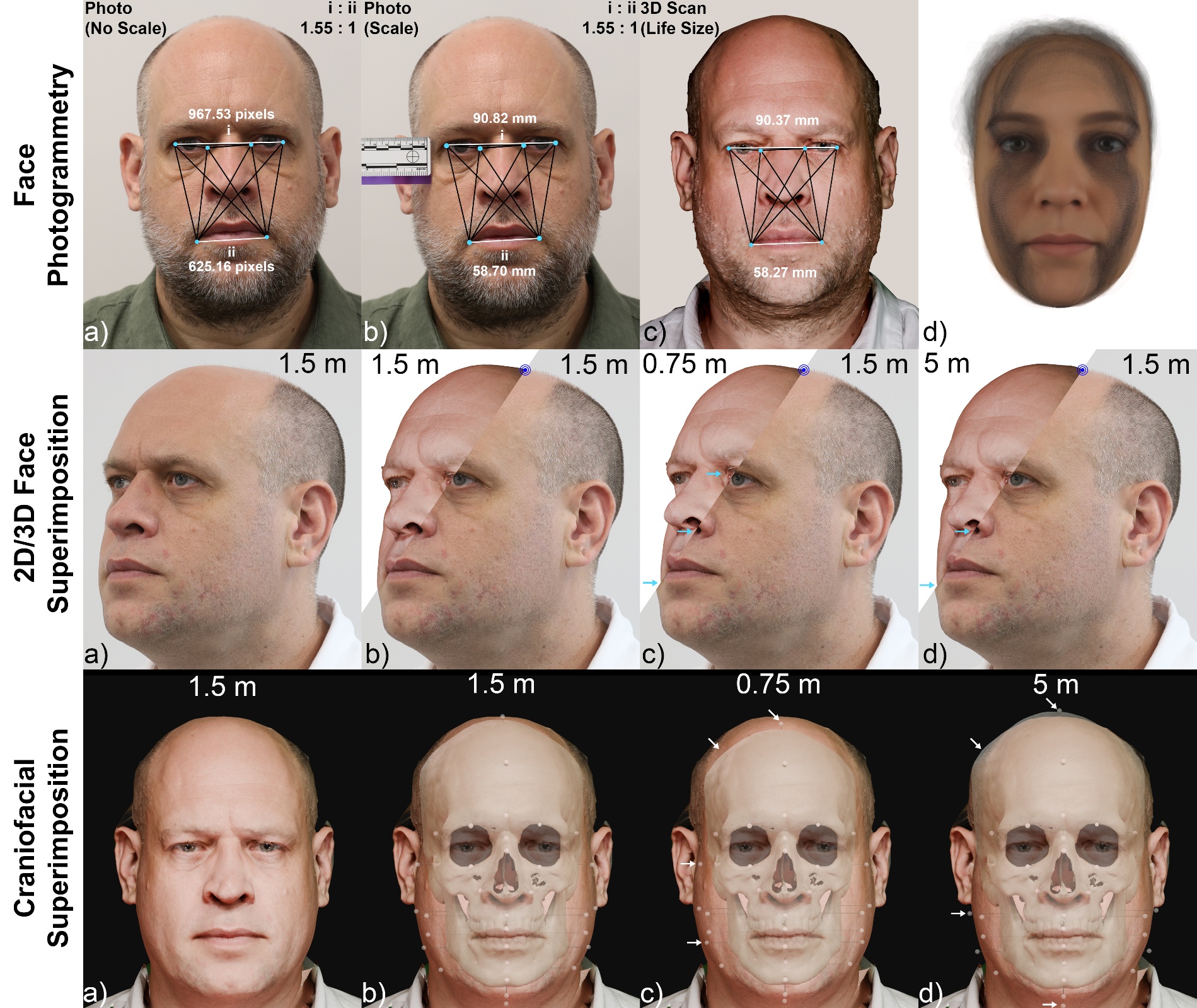


Figure 1. Examples of forensic science relevance of facial dimensions, and the importance of scales for measurements from photographs in forensic science applications: face photogrammetry [1, 2] (Row 1); 2D/3D superimposition [3-6] (Row 2); and craniofacial superimposition [7-9] (Row 3).

Row 1: a) When no scale is present, only ratios of feature dimensions in pixels are possible [1]; b) when a scale at the correct focus distance is present, both life size measurements and ratios can be obtained as illustrated; c) comparative 3D Vectra® M3 scan with corresponding metrics indicated; and d) the mean zone (black highlight) where facial feature measurement in photogrammetry is valid no matter what focus distance applies for the image (= relative parallel zone [RPZ] after [10, 11]). Note that all dimensions in (a) and (b) fall within the RPZ. Image (d) reproduced from [10] with permission by Elsevier.

Row 2: a) 2D photograph of a subject at 1.5 m focus distance; b) 2D/3D superimposition, after [3-6], using a focus distance matched 2D render of a 3D Vectra® M3 scan (focus distance = 1.5 m); c) malalignment of same face when a mismatched focus distance (-0.75 m) is used for the 2D rendered 3D scan; and d) malalignment of same face when a mismatched focus distance (+3.5 m) is used for the 2D rendered 3D scan. Blue arrows highlight mismatched face anatomy at mismatched focus distances. c) and d) highlight the importance of using a scale to correctly set the focus distance for measurements in the 2D/3D face superimposition procedure. Two-dimensional renders of the 3D scan at different focus distances in this image row were generated in Blender.

Row 3: a) target face for craniofacial superimposition (1.5 m focus distance); b) craniofacial superimposition using a 3D surface render of a skull at a 1.5 m focus distance, highlighting the importance of a correctly set focus distance using a within photograph scale; c) malalignment of the same skull (as in b) when mismatched focus distance (-0.75 m) is used; and d) malalignment of same skull (as in b) when mismatched focus distance (+3.5 m) is used. White arrows highlight mismatched anatomy at mismatched focus distances owing to improper or no scale use. c) and d) highlight the importance of using a scale to correctly set the focus distance for measurements in the craniofacial superimposition procedure. 2D renders of the skull generated in Blender. The white pins on the skull represent with ground truth tissue thicknesses for the target individual.

References

1. Kleinberg KF, Vanezis P, Burton AM (2007) Failure of anthropometry as a facial identification technique using high-quality photographs. Journal of Forensic Sciences 52: 779-83. https://doi.org/10.1111/j.1556-4029.2007.00458.x

2. Porter G, Doran G (2000) An anatomical and photographic technique for forensic facial identification. Forensic Science International 114: 97-105.

3. Yoshino M, Matsuda H, Kubota S, Imaizumi K, Miyasaka S (2000) Computer-assisted facial image identification system using a 3-D physiognomic range finder. Forensic Science International 109: 225-37. https://doi.org/

4. Lynnerup N, Clausen M-L, Kristoffersen AM, Steglich-Arnholm H (2009) Facial recognition and laser surface scan: a pilot study. Forensic Sci Med Pathol 5: 167-73. https://doi.org/10.1007/s12024-009-9094-8

5. De Angelis D, Sala R, Cantatore A, Grandi M, Cattaneo C (2009) A new computer-assisted technique to aid personal identification. International Journal of Legal Medicine 123: 351-6. https://doi.org/10.1007/s00414-008-0311-x

6. Buck U, Naether S, Kreutz K, Thali M (2011) Geometric facial comparisons in speed-check photographs. Int J Legal Med 125: 785-90. https://doi.org/10.1007/s00414-010-0518-5

7. Glaister J, Brash JC. (1937) Medico-legal aspects of the Ruxton case. William Wood and Co. Baltimore.

8. Taylor JA, Brown KA. (1998) Superimposition techniques. In: Clement JG, Ranson DL, eds. Craniofacial Identification in Forensic Medicine. Hodder Arnold London. pp. 151-64.

9. Yoshino M. (2012) Craniofacial superimposition. In: Wilkinson CM, Rynn C, eds. Craniofacial Identification. Cambridge University Press Cambridge. pp. 238-53.

10. Stephan CN, Armstrong B (2021) Scientific estimation of the subject-to-camera distance from facial photographs for craniofacial superimposition. Forensic Science International: Reports 4: 100238. https://doi.org/10.1016/j.fsir.2021.100238

11. Lan Y, Cai D. (1993) Technical advances in skull-to-photo superimposition. In: İşcan MY, Helmer RP, eds. Forensic Analysis of the Skull. Wiley-Liss New York. pp. 119-29.

Title: Mean Human Corneal Diameter and Palpebral Fissure Lengths as Scales for Forensic Analysis of Photographed Faces: An Analytical Review

Journal Name: International Journal of Legal Medicine

Author Names: Sean S. Healy & Carl N. Stephan

Affiliation: Laboratory for Human Craniofacial and Skeletal Identification (HuCS-ID Lab), School of Biomedical Sciences, The University of Queensland, Brisbane, Australia, 4072.

Corresponding Author Email: sean.healy@uq.net.au
