# Supplementary material for: Mean human corneal diameter and palpebral fissure lengths as scales for forensic analysis of photographed faces: an analytical review*
Source: Int J Legal Med. 2026 Feb 23;140(3):1529–46. doi: 10.1007/s00414-026-03733-0 (PMC13161299; doi:10.1007/s00414-026-03733-0)
Supplement: Supplementary file 2 — Supplementary Material 2 [file 414_2026_3733_MOESM2_ESM.docx]

**Supplementary Material 2**

**Cornea Anatomy in Brief**

The cornea forms the anterior pole of the eyeball and borders the anterior chamber of the eye, anterior to the iris. It forms a bulbous like protrusion in transverse section (Fig. 1) and merges with the white sclera that encircles it. This junction between the two structures, the cornea and the sclera, is termed the limbus [1]. In some individuals, the limbus is very clearly manifested a distinct dark ring around the iris that may additionally be referred to as a ‘limbal ring’ or a ‘dark limbus’ [2].


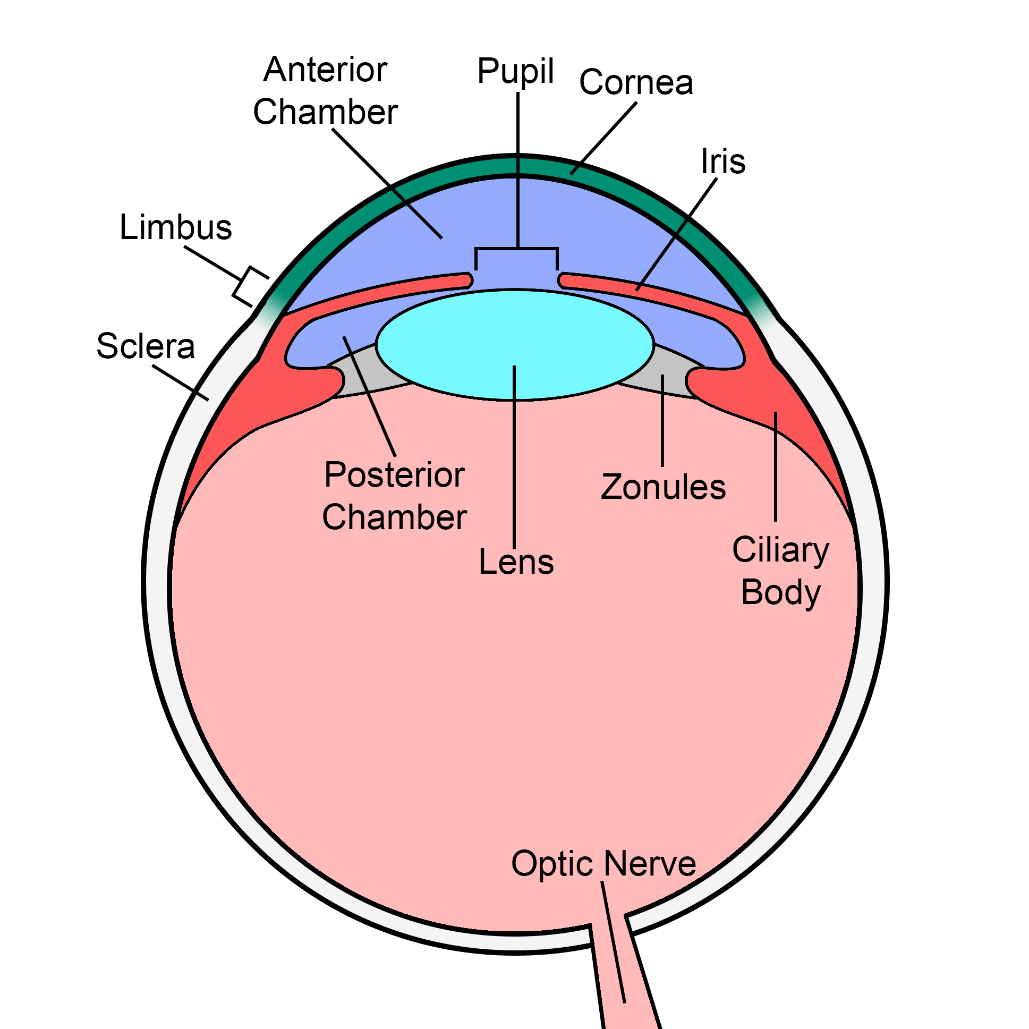


**Figure 1.** Anatomical components of the human eye.

**References**

1. Van Buskirk EM (1989) The anatomy of the limbus. Eye 3:101-8. https://doi.org/10.1038/eye.1989.16

2. Shyu BP, Wyatt HJ (2009) Appearance of the human eye: Optical contributions to the “limbal ring”. Optom Vis Sci 86:E1069-E77. https://doi.org/10.1097/OPX.0b013e3181b4f010

Title: Mean Human Corneal Diameter and Palpebral Fissure Lengths as Scales for Forensic Analysis of Photographed Faces: An Analytical Review

Journal Name: International Journal of Legal Medicine

Author Names: Sean S. Healy & Carl N. Stephan

Affiliation: Laboratory for Human Craniofacial and Skeletal Identification (HuCS-ID Lab), School of Biomedical Sciences, The University of Queensland, Brisbane, Australia, 4072.

Corresponding Author Email: sean.healy@uq.net.au
