# Supplementary material for: Mean human corneal diameter and palpebral fissure lengths as scales for forensic analysis of photographed faces: an analytical review*
Source: Int J Legal Med. 2026 Feb 23;140(3):1529–46. doi: 10.1007/s00414-026-03733-0 (PMC13161299; doi:10.1007/s00414-026-03733-0)
Supplement: Supplementary file 3 — Supplementary Material 3 [file 414_2026_3733_MOESM3_ESM.docx]

**Supplementary Material 3**

**Mean Adult Corneal Diameter statistics with References**

**Table 1:** Mean adult corneal diameter by instrument and ordered by pooled study sample size (number of eyes)

| **Instrument** | **Weighted mean (mm)** | **Combined SD (mm)** | **Number of eyes (n)** | **Number of studies (n)** | **Study Reference Numbers** |
| --- | --- | --- | --- | --- | --- |
| IOLMaster 700 | 11.80 | 0.48 | 194,755 | 80 | [1-78] |
| Lenstar LS900 | 12.03 | 0.51 | 30,282 | 32 | [8, 62, 79-108] |
| Pentacam | 11.58 | 0.41 | 14,667 | 15 | [19, 57, 73, 109-121] |
| IOLMaster | 11.97 | 0.41 | 14,578 | 10 | [122-131] |
| Pentacam AXL | 11.61 | 0.39 | 13,058 | 16 | [34, 40, 43, 50, 52, 60, 65, 132-140] |
| Pentacam HR | 11.74 | 0.51 | 9,270 | 24 | [27, 33, 67, 141-161] |
| IOLMaster 500 | 11.77 | 0.50 | 8,921 | 19 | [58, 136, 138, 162-178] |
| Anterion SS-OCT | 11.88 | 0.49 | 2,803 | 22 | [1, 36, 45, 60, 65, 67, 68, 70, 77, 100, 153, 173, 179-188] |
| Nidek OPD-Scan III | 11.80 | 0.68 | 2,147 | 6 | [6, 8, 19, 81, 107, 189] |
| Tomey OA-2000 | 11.69 | 0.52 | 2,019 | 7 | [20, 62, 162, 190-193] |
| Orbscan 3 | 11.50 | 0.40 | 1,815 | 1 | [194] |
| Argos SS-OCT | 12.22 | 0.61 | 1,248 | 8 | [44, 60, 62, 132, 195-198] |
| Nidek AL-Scan | 11.88 | 0.42 | 1,072 | 7 | [18, 62, 199-203] |
| Topcon Aladdin | 11.54 | 0.46 | 889 | 5 | [43, 62, 195, 204, 205] |
| Galilei G6 | 12.12 | 0.32 | 683 | 6 | [139, 140, 153, 172, 206, 207] |
| Tomey Casia 2 AS-OCT | 11.62 | 0.61 | 667 | 7 | [27, 208-213] |
| EyeStar 900 | 12.06 | 0.42 | 645 | 4 | [44, 182, 214, 215] |
| Schwind Sirius | 11.89 | 0.54 | 584 | 8 | [11, 104, 151, 189, 215-218] |
| Visante AS-OCT | 11.85 | 0.47 | 566 | 1 | [219] |
| Orbscan II | 11.65 | 0.40 | 557 | 6 | [6, 50, 56, 189, 220, 221] |
| Schwind MS-39 | 11.88 | 0.43 | 429 | 4 | [188, 215, 222, 223] |
| Quantel AB Scan Biometer | 11.45 | 0.89 | 400 | 1 | [224] |
| Suoer SW-9000 | 11.64 | 0.40 | 223 | 3 | [192, 222, 225] |
| Catalys femtosecond SD-OCT | 11.40 | 0.48 | 199 | 2 | [68, 226] |
| Colombo IOL 2 | 12.23 | 0.40 | 159 | 1 | [13] |
| Alcon Verion | 11.91 | 0.49 | 116 | 1 | [49, 227] |
| Revo 80 | 12.85 | 0.61 | 144 | 1 | [137] |
| Galilei G4 | 12.15 | 0.40 | 142 | 1 | [107] |
| Cirrus HD-OCT | 12.02 | 0.51 | 122 | 2 | [57, 228] |
| ZW-30 | 11.95 | 0.42 | 82 | 1 | [229] |
| Nidek ARK-1 | 12.01 | 0.44 | 65 | 1 | [97] |
| Cassini Colour LED | 12.65 | 0.52 | 49 | 1 | [67] |
| Topcon MYAH | 12.15 | 0.36 | 40 | 1 | [106] |
| Scansys TA517 | 12.06 | 0.35 | 38 | 1 | [217] |
| DRI-OCT Triton | 12.40 | 0.48 | 33 | 1 | [189] |
| Svision VG200 | 11.64 | 0.39 | 32 | 1 | [230] |
| MR-6000 | 11.46 | 0.32 | 30 | 1 | [56] |
| BQ 900 | 11.97 | 0.59 | 20 | 1 | [152] |
| **Grand Pool** | **11.84** | **0.79** | **296,887** | **234** | **See rows above** |

**References**

1. McLintock C, Niyazmand H, Seo S, Barrett G, Nilagiri VK, McKelvie J (2022) Agreement between 2 SS-OCT biometry devices. J Cataract Refract Surg 48:1107-12. https://doi.org/10.1097/j.jcrs.0000000000000942

2. Mursch-Edlmayr AS, Pomberger LJ, Hermann P, Wagner H, Beka S, Waser K, Wendelstein J, Podkowinski D, Laubichler P, Siska R, Bolz M (2021) Prospective comparison of apex-centered vs standard pupil-centered femtosecond laser–assisted capsulotomy in cataract surgery. J Cataract Refract Surg 47:606-11. https://doi.org/10.1097/j.jcrs.0000000000000496

3. Langenbucher A, Szentmáry N, Cayless A, Casaza M, Weisensee J, Hoffmann P, Wendelstein J (2022) Surgically induced astigmatism after cataract surgery – A vector analysis. Curr Eye Res 47:1279-87. https://doi.org/10.1080/02713683.2022.2052108

4. Schranz M, Reumüller A, Kostolna K, Novotny C, Schartmüller D, Abela-Formanek C (2023) Refractive outcome and lens power calculation after intrascleral intraocular lens fixation: A comparison of three-piece and one-piece intrascleral fixation technique. Eye Vis (Lond) 10:29. https://doi.org/10.1186/s40662-023-00341-6

5. Langenbucher A, Cayless A, Szentmáry N, Weisensee J, Wendelstein J, Hoffmann P (2021) Prediction of total corneal power from measured anterior corneal power on the IOLMaster 700 using a feedforward shallow neural network. Acta Ophthalmol 100:e1080-e7. https://doi.org/10.1111/aos.15040

6. Cruz S, Valenzuela F, Stoppel J, Maul E, Gibbons A (2021) Comparison of horizontal corneal diameter measurements using Orbscan IIz, OPD Scan III, and IOLMaster 700. Eye Contact Lens 47:533-8. https://doi.org/10.1097/ICL.0000000000000786

7. Feng X, Wang Y, Liang J, Xu Y, Ortega-Usobiaga J, Cao D (2021) Analysis of lens thickness distribution based on swept-source optical coherence tomography (SS-OCT). J Ophthalmol 2021:4717996. https://doi.org/10.1155/2021/4717996

8. Bao T, Wang L, Liu C, Yang Y, Pang Y (2023) Analysis of biometric parameters of cataract eyes measured with optical biometer Lenstar LS900, IOL Master 700, and OPD-SCAN III. Photodiagnosis Photodyn Ther 43:103646. https://doi.org/10.1016/j.pdpdt.2023.103646

9. Wang Z, Song Y, Yang W, Li D, Chen W, Zhao Q, Liu Q, Zhai C (2022) Comparing standard keratometry and total keratometry before and after myopic corneal refractive surgery with a swept-source OCT biometer. Front Med 9:928027. https://doi.org/10.3389/fmed.2022.928027

10. Chang J, Wang L, Jiang C, Song Z, Lu P (2024) Predicting the postoperative intraocular lens position based on IOL Master 700 biometry, compared with results from the anterior segment analysis system. Graefes Arch Clin Exp Ophthalmol 262:113-9. https://doi.org/10.1007/s00417-023-06175-1

11. Lu W, Li Y, Savini G, Song B, Hu Q, Wang Q, Bao F, Huang J (2019) Comparison of anterior segment measurements obtained using a swept-source optical coherence tomography biometer and a Scheimpflug–Placido tomographer. J Cataract Refract Surg 45:298-304. https://doi.org/10.1016/j.jcrs.2018.10.033

12. Chen Y, Yang P, Li W, Huo L (2024) Ocular characteristics and outcomes of phacoemulsification in patients with cataract after renal transplantation. Int Ophthalmol 44:255. https://doi.org/10.1007/s10792-024-03161-2

13. Sun B, Ye Y, Zhao J, Zhou X, Niu L (2024) Comparison of axial length and anterior segment parameters of patients with myopia measured using 2 fourier-domain optical coherent biometry devices. BMC Ophthalmol 24:289. https://doi.org/10.1186/s12886-024-03546-y

14. Shi R, Ma D, Zeng Q, Hua Z, Shen W, Cai L, Yang J (2024) Rotational stability of monofocal and diffractive multifocal toric intraocular lens with identical design and material: A propensity score based prospective comparative study. BMC Ophthalmol 24:72. https://doi.org/10.1186/s12886-024-03281-4

15. Yang H, Qian D, Chan G, Wang J, Sun X, Chen Y (2023) Influence of miosis and laser peripheral iridotomy on intraocular lens power calculation in patients with primary angle closure disease. Eye 37:2744-52. https://doi.org/10.1038/s41433-023-02408-z

16. Ma D, Han X, Hua Z, Shen J, Zhang L, Qiu T, Luo J, Cai L, Yang J (2022) Propensity-matched comparison of postoperative stability and visual outcomes of toric intraocular lens with or without a capsular tension ring and updated meta-analysis. Graefes Arch Clin Exp Ophthalmol 261:989-98. https://doi.org/10.1007/s00417-022-05851-y

17. Wei L, He W, Meng J, Qian D, Lu Y, Zhu X (2021) Evaluation of the white-to-white distance in 39,986 Chinese cataractous eyes. Invest Ophthalmol Vis Sci 62:7. https://doi.org/10.1167/iovs.62.1.7

18. Chan TCY, Wan KH, Tang FY, Wang YM, Yu M, Cheung C (2020) Repeatability and agreement of a swept-source optical coherence tomography–based biometer IOLMaster 700 versus a Scheimpflug imaging–based biometer AL-Scan in cataract patients. Eye Contact Lens 46:35-45. https://doi.org/10.1097/ICL.0000000000000603

19. Zhu Q-J, Zhu W-J, Chen W-J, Ma L, Yuan Y (2022) A prediction model for sulcus-to-sulcus diameter in myopic eyes: A 1466-sample retrospective study. BMC Ophthalmol 22:307. https://doi.org/10.1186/s12886-022-02535-3

20. Yeu E (2019) Agreement of ocular biometry measurements between 2 biometers. J Cataract Refract Surg 45:1130-4. https://doi.org/10.1016/j.jcrs.2019.03.016

21. Di Y, Fang H, Luo Y, Li Y, Xu Y (2024) Predicting implantable collamer lens vault using machine learning based on various preoperative biometric factors. Transl Vis Sci Technol 13:8. https://doi.org/10.1167/tvst.13.1.8

22. Zhang X, Ma J, Li L, Gan L, He H, Shao E, Guo X, Zhu H, You H, Zhong Y, Xing B (2022) Elevated IGF-1 and GH levels are correlated with a thicker iris and wider anterior chamber angle in treatment-naïve acromegaly patients. Invest Ophthalmol Vis Sci 63:27. https://doi.org/10.1167/iovs.63.11.27

23. Chen X, Zhang D, Liu Z, Liu Y, Cai H, Wu Q, Zhang Y (2021) Effect of implantable collamer lens on anterior segment measurement and intraocular lens power calculation based on IOLMaster 700 and Sirius. J Ophthalmol 2021:8988479. https://doi.org/10.1155/2021/8988479

24. Li Q, Liu X, Yang J, Dai Y, Li W (2024) Comparison of Barrett TK Universal II and Barrett Universal II TCRP formulas in power calculations for 3 presbyopia-correcting intraocular lenses. Clin Ophthalmol 18:1457-65. https://doi.org/10.2147/OPTH.S461195

25. Xi W, Yang M, Wan J, Wang Y, Qiao Y, Huang X, Liu X, Fan N, Liu S, Zeng K, Chen S (2022) Effect of pupil dilation on biometry measurements and intraocular lens power in eyes with high myopia. Front Med 9:963599. https://doi.org/10.3389/fmed.2022.963599

26. Zhang J, Jin A, Han X, Chen Z, Diao C, Zhang Y, Liu X, Xu F, Liu J, Qiu X, Tan X, Luo L, Liu Y (2024) The LISA-PPV formula: An ensemble artificial Iintelligence-based thick intraocular lens calculation formula for vitrectomized eyes. Am J Ophthalmol 262:237-45. https://doi.org/10.1016/j.ajo.2024.02.037

27. Ma S, Li C, Sun J, Yang J, Wen K, Chen X-T, Zhao F-Y, Gao R-Y, Tian F (2024) Comparison of ocular biometric parameters between two swept-source optical coherence tomography devices and Scheimpflug tomography in patients with cataract. Int J Ophthalmol 17:1437-46. https://doi.org/10.18240/ijo.2024.08.08

28. Huang J, Zhao Y, Savini G, Yu G, Yu J, Chen Z, Tu R, Zhao Y (2020) Reliability of a new swept-source optical coherence tomography biometer in healthy children, adults, and cataract patients. J Ophthalmol 2020:8946364. https://doi.org/10.1155/2020/8946364

29. Gu X, Duan Q, He J, Zhang T, Tang L, Ma B (2024) Distribution and associations of anterior lens zonules lengths in patients with cataract. Graefes Arch Clin Exp Ophthalmol 262:2515-23. https://doi.org/10.1007/s00417-024-06379-z

30. Zheng S, Wang D, Huang Z, Chen L, Hu H, Hu M, Tang Z, Zuo C, Lin M (2023) Distribution of ultrasound cyclo plasty probe models in Chinese patients with glaucoma. Int Ophthalmol 43:4435-41. https://doi.org/10.1007/s10792-023-02818-8

31. Zhang J, Chen F, Han X, Qiu X, Liu Z, Chen X, Jin G, Qu B, Yao H, Ye Y, Yu K, Tan X, Luo L (2023) Vault height is a key predictive factor for anterior segment measurement error by IOLMaster 700 in eyes with phakic intraocular lens. Transl Vis Sci Technol 12:16. https://doi.org/10.1167/tvst.12.9.16

32. Mirshahi A, Ponto KA (2019) Changes in pupil area during low-energy femtosecond laser-assisted cataract surgery. J Ophthalmic Vis Res 14:251-6. https://doi.org/10.18502/jovr.v14i3.4780

33. Karmiris E, Gartaganis PS, Ntravalias T, Manousakis E, Giannakis I, Chalkiadaki E (2022) Agreement between swept-source optical biometry and Scheimpflug-based tomography in eyes with previous myopic refractive surgery. Saudi J Ophthalmol 36:229-36. https://doi.org/10.4103/sjopt.sjopt_138_21

34. Chalkiadaki E, Gartaganis PS, Ntravalias T, Giannakis I, Manousakis E, Karmiris E (2022) Agreement in anterior segment measurements between swept-source and Scheimpflug-based optical biometries in keratoconic eyes: A pilot study. Ther Adv Ophthalmol 14. https://doi.org/10.1177/25158414211063283

35. Sharma A, Batra A (2021) Assessment of precision of astigmatism measurements taken by a swept-source optical coherence tomography biometer - IOLMaster 700. Indian J Ophthalmol 69:1760–5. https://doi.org/10.4103/ijo.IJO_2776_20

36. Panda A, Nanda A, Sahoo K (2022) Comparison of ocular biometry and refractive outcome between ANTERION and IOL Master 700. Indian J Ophthalmol 70:1594-8. https://doi.org/10.4103/ijo.IJO_2433_21

37. Brar S, Ganesh S, Karegowda M (2024) Clinical outcomes and rotational stability after implantation of a monofocal toric intraocular lens with textured haptics in normal vs high axial lengths. J Cataract Refract Surg 50:718-23. https://doi.org/10.1097/j.jcrs.0000000000001429

38. Tiwari P, Raj A, Nishant P (2024) Estimation of angle parameters by ASOCT and biometric parameters by optical biometer in eyes with occludable angles and open angles. Int Ophthalmol 44:160. https://doi.org/10.1007/s10792-024-03086-w

39. Zarei R, Zamani MH, Eslami Y, Fakhraei G, Tabatabaei M, Esfandiari AR (2022) Comparing corneal biomechanics and intraocular pressure between healthy individuals and glaucoma subtypes: A cross-sectional study. Ann Med Surg (Lond) 82. https://doi.org/10.1016/j.amsu.2022.104677

40. Salouti R, Kamalipour A, Masihpour N, Zamani M, Ghoreyshi M, Salouti K, Nowroozzadeh MH (2020) Effect of photorefractive keratectomy on agreement of anterior segment variables obtained by a swept-source biometer vs a Scheimpflug-based tomographer. J Cataract Refract Surg 46:1229-35. https://doi.org/10.1097/j.jcrs.0000000000000252

41. Vega Y, Gershoni A, Achiron A, Tuuminen R, Weinberger Y, Livny E, Nahum Y, Bahar I, Elbaz U (2021) High agreement between Barrett Universal II calculations with and without utilization of optional biometry parameters. J Clin Med 10:542. https://doi.org/10.3390/jcm10030542

42. Wasser LM, Tsessler M, Weill Y, Zadok D, Abulafia A (2022) Ocular biometric characteristics measured by swept-source optical coherence tomography in individuals undergoing cataract surgery. Am J Ophthalmol 233:38-47. https://doi.org/10.1016/j.ajo.2021.06.032

43. Savini G, Lupardi E, Hoffer KJ, Aramberri J, Schiano-Lomoriello D (2022) Corneal diameter measurements by 3 optical biometers and their effect on phakic intraocular lens sizing. J Cataract Refract Surg 48:1292-6. https://doi.org/10.1097/j.jcrs.0000000000000976

44. Galzignato A, Lupardi E, Hoffer KJ, Barboni P, Schiano-Lomoriello D, Savini G (2023) Repeatability of new optical biometer and agreement with 2 validated optical biometers, all based on SS-OCT. J Cataract Refract Surg 49:5-10. https://doi.org/10.1097/j.jcrs.0000000000001023

45. Kato Y, Kojima T, Tamaoki A, Ichikawa K, Tamura K, Ichikawa K (2022) Refractive prediction error in cataract surgery using an optical biometer equipped with anterior segment OCT. J Cataract Refract Surg 48:429-34. https://doi.org/10.1097/j.jcrs.0000000000000781

46. Danjo Y, Ohji R, Maeno S (2023) Lower refractive prediction accuracy of total keratometry using intraocular lens formulas loaded onto a swept-source optical biometer. Graefes Arch Clin Exp Ophthalmol 261:137-46. https://doi.org/10.1007/s00417-022-05777-5

47. Tanaka T, Nishitsuka K, Obata H (2024) Correlation of ocular biometry with axial length in E=elderly Japanese. Clin Ophthalmol 18:351-60. https://doi.org/10.2147/OPTH.S446031

48. Teshigawara T, Meguro A, Mizuki N (2020) Influence of pupil dilation on the Barrett Universal II (new generation), Haigis (4th generation), and SRK/T (3rd generation) intraocular lens calculation formulas: A retrospective study. BMC Ophthalmol 20:299. https://doi.org/10.1186/s12886-020-01571-1

49. Moreno-Páramo E, García-Arroyo S, Ortiz-Ramirez GY, Garza-León M, Gonzalez-Salinas R (2022) Agreement between the swept-source optical coherence tomography and the image-guided system for biometry assessment in cataract surgery. Semin Ophthalmol 37:324-9. https://doi.org/10.1080/08820538.2021.1965172

50. Ang RET, Reyes EKF, Ayuyao FAJ, Umali MIN, Cruz EM (2022) Comparison of white-to-white measurements using four devices and their determination of ICL sizing. Eye Vis (Lond) 9:36. https://doi.org/10.1186/s40662-022-00308-z

51. Stopyra W, Voytsekhivskyy O, Grzybowski A (2024) Accuracy of 20 intraocular lens power calculation formulas in medium-long eyes. Ophthalmol Ther 13:1893-907. https://doi.org/10.1007/s40123-024-00954-7

52. Oleszko AA, Marek JJ, Muzyka‐woźniak MM (2021) Horizontal and anterior chamber diameter for phakic intraocular lens sizing. Clin Exp Optom 104:62-8. https://doi.org/10.1111/cxo.13101

53. Izdebska J, Bombuy Gimenez J, Przybek-Skrzypecka J, Szaflik Jacek P, Skrzypecki J (2024) Utilization of the Reinstein ICL sizing formula with hand-held ultrasound biomicroscopy measurements. J Refract Surg 40:e142-e7. https://doi.org/10.3928/1081597X-20240206-01

54. Jeon S, Choi A (2021) Risk factor for transient hyperopic refractive outcome at acute postoperative period after panoptix intraocular lens implantation. Clin Ophthalmol 15:2499-503. https://doi.org/10.2147/OPTH.S318286

55. Kim S, Oh R, Kim MK, Yoon CH (2023) SS-OCT-based ocular biometry in an adult Korean population with cataract. J Cataract Refract Surg 49:453-9. https://doi.org/10.1097/j.jcrs.0000000000001135

56. Kim BY, Jun I (2022) Comparison of anterior segment measurements with a new multifunctional unit and five other devices. Korean J Ophthalmol 36:338-49. https://doi.org/10.3341/kjo.2022.0025

57. Calvo-Sanz JA, Poyales F, Zhou Y, Arias-Puente A, Garzón N (2022) Agreement between the biometric measurements used to calculate the size of the implantable collamer lenses measured with four different technologies. Indian J Ophthalmol 70:1586-92. https://doi.org/10.4103/ijo.IJO_2217_21

58. Tañá-Rivero P, Tello-Elordi C, Orts-Vila P, Tañá-Sanz P, Tañá-Sanz S (2023) Agreement of corneal diameter and anterior chamber depth measurements with the IOLMaster 500 and the IOLMaster 700 optical biometers in myopic eyes. Clin Ophthalmol 17:1245-53. https://doi.org/10.2147/OPTH.S409132

59. Fernández-Rosés J, Lamarca J, Piñero DP, Barraquer RI (2021) Intraocular lens power calculation in virgin eyes: Accuracy of the Barrett Universal II formula and a ray tracing software. Eur J Ophthalmol 32:2954-60. https://doi.org/10.1177/11206721211065552

60. Tañá-Rivero P, Aguilar-Córcoles S, Tañá-Sanz P, Tañá-Sanz S, Montés-Micó R (2023) Axial length acquisition success rates and agreement of four optical biometers and one ultrasound biometer in eyes with dense cataracts. Eye Vis (Lond) 10:35. https://doi.org/10.1186/s40662-023-00352-3

61. Albarrán-Diego C, Poyales F, López-Artero E, Garzón N, García-Montero M (2022) Interocular biometric parameters comparison measured with swept-source technology. Int Ophthalmol 42:239-51. https://doi.org/10.1007/s10792-021-02020-8

62. Montés-Micó R (2022) Evaluation of 6 biometers based on different optical technologies. J Cataract Refract Surg 48:16-25. https://doi.org/10.1097/j.jcrs.0000000000000690

63. Carmona González D, Palomino Bautista C (2021) Accuracy of a new intraocular lens power calculation method based on artificial intelligence. Eye 35:517-22. https://doi.org/10.1038/s41433-020-0883-3

64. Jiménez-García M, Segura-Calvo FJ, Puzo M, Castro-Alonso FJ, Zaragoza UFRAG (2024) Biometric description of 34 589 eyes undergoing cataract surgery: sex differences. J Cataract Refract Surg 50:668-75. https://doi.org/10.1097/j.jcrs.0000000000001435

65. Tañá-Sanz P, Rodríguez-Carrillo MD, Ruiz-Santos M, Montés-Micó R, Ruiz-Mesa R, Tañá-Rivero P (2021) Agreement of predicted intraocular lens power using swept-source optical coherence tomography and partial coherence interferometry. Expert Rev Med Devices 18:1219-34. https://doi.org/10.1080/17434440.2021.2008908

66. Fernández J, Rodríguez-Vallejo M, Poyales F, Burguera N, Garzón N (2020) New method to assess the accuracy of intraocular lens power calculation formulas according to ocular biometric parameters. J Cataract Refract Surg 46:849-56. https://doi.org/10.1097/j.jcrs.0000000000000165

67. Tañá-Rivero P, Aguilar-Córcoles S, Rodríguez-Prats JL, Montés-Micó R, Ruiz-Mesa R (2021) Agreement of white-to-white measurements with swept-source OCT, Scheimpflug and color LED devices. Int Ophthalmol 41:57-65. https://doi.org/10.1007/s10792-020-01552-9

68. Tañá-Sanz P, Ruiz-Santos M, Rodríguez-Carrillo MD, Aguilar-Córcoles S, Montés-Micó R, Tañá-Rivero P (2021) Agreement between intraoperative anterior segment spectral-domain OCT and 2 swept-source OCT biometers. Expert Rev Med Devices 18:387-93. https://doi.org/10.1080/17434440.2021.1905518

69. Wendelstein JA, Rothbächer J, Heath M, McDonald MC, Hoffmann PC, Cooke DL, Seiler TG, Langenbucher A, Riaz KM (2023) Influence and predictive value of optional parameters in new-generation intraocular lens formulas. J Cataract Refract Surg 49:795-803. https://doi.org/10.1097/j.jcrs.0000000000001207

70. Pfaeffli OA, Weber A, Hoffer KJ, Savini G, Baenninger PB, Thiel MA, Taroni L, Müller L (2022) Agreement of intraocular lens power calculation between 2 SS-OCT–based biometers. J Cataract Refract Surg 48:534-41. https://doi.org/10.1097/j.jcrs.0000000000000788

71. Srivannaboon S, Chirapapaisan C (2019) Comparison of refractive outcomes using conventional keratometry or total keratometry for IOL power calculation in cataract surgery. Graefes Arch Clin Exp Ophthalmol 257:2677-82. https://doi.org/10.1007/s00417-019-04443-7

72. Ozcaliskan S, Yenerel NM (2019) The effect of cycloplegia on biometric measurements using swept‐source optical coherence tomography‐based biometry. Clin Exp Optom 102:501-5. https://doi.org/10.1111/cxo.12888

73. Güçlü H, Akaray İ, Kaya S, Sattarpanah S, Çınar AC, Sakallıoğlu K, Korkmaz S, Gürlü V (2021) Agreement of anterior segment parameters between schiempflug topography and swept-Source optic coherence based optic biometry in keratoconus and healthy subjects. Eye Contact Lens 47:539-45. https://doi.org/10.1097/ICL.0000000000000787

74. Reinstein DZ, Archer TJ, Vida RS, Piparia V, Potter JG (2022) New sizing parameters and model for predicting postoperative vault for the implantable collamer lens posterior chamber phakic intraocular lens. J Refract Surg 38:272-9. https://doi.org/10.3928/1081597x-20220302-01

75. Voytsekhivskyy OV (2023) Accuracy of the VRF and VRF-G intraocular lens power calculation formulas using swept-source optical coherence tomography biometry. Clin Ophthalmol 17:3663-72. https://doi.org/10.2147/OPTH.S439287

76. Lam BC, Weiss M, Jing F, Zhu C, Johnson DA, Kheirkhah A (2022) Comparison of ocular biometric parameters between Hispanic and Non-Hispanic ethnicities in White Adults undergoing cataract surgery. Eye Contact Lens 48:391-5. https://doi.org/10.1097/ICL.0000000000000907

77. Agard E, Levron A, Billant J, Douma I, Dot C (2024) Comparison of refractive outcomes obtained with two swept-source OCT-based optical biometers after cataract surgery: A study of 152 eyes. J Fr Ophtalmol 47:104186. https://doi.org/10.1016/j.jfo.2024.104186

78. Zhang J, Han X, Zhang M, Liu Z, Chen X, Qiu X, Lin H, Li J, Liu B, Zhang C, Wei Y, Jin G, Tan X, Luo L (2022) Predicting the risk of clinically significant intraocular lens tilt and decentration in vitrectomized eyes. J Cataract Refract Surg 48:1318-24. https://doi.org/10.1097/j.jcrs.0000000000000997

79. Li P, Tu Y, Chen X, Song Y, Guan H (2019) Clinical outcomes of steep-axis one-handed phacoemulsification under the guidance of a Verion image-guided system. J Ophthalmol 2019:7182324. https://doi.org/10.1155/2019/7182324

80. Zhang F, Li S, Huo D, Li Q (2022) Predictors of femtosecond laser–assisted arcuate keratotomy efficacy for astigmatism correction in cataract surgery. J Refract Surg 38:480-6. https://doi.org/10.3928/1081597X-20220609-01

81. Bao T, Yin L, Liu C, Pang Y, Zhang G, Yang Y, Zhang B (2023) Agreement of anterior segment measurements between LenStar LS 900 optical biometer and OPD Scan III wavefront aberrometer devices in eyes with cataract. Photodiagnosis Photodyn Ther 41:103207. https://doi.org/10.1016/j.pdpdt.2022.103207

82. Cui Y, Yang X, Zhang G, Guo H, Zhang M, Zhang L, Zeng J, Liu Q, Zhang L, Meng Q (2019) Intraocular pressure in general and diabetic populations from southern China: The dongguan eye study. Invest Ophthalmol Vis Sci 60:761-9. https://doi.org/10.1167/iovs.18-25247

83. Gao R, Liu J, Zhou X, Huang L, Huang W, Xue Y, Wang F, Gong S, Wu R, Wang Y (2023) Influence of pilocarpine eyedrops on the ocular biometric parameters and intraocular lens power calculation. J Ophthalmol 2023:7680659. https://doi.org/10.1155/2023/7680659

84. Li P-P, Huang Y-M, Cai Q, Huang L-L, Song Y, Guan H-J (2019) Effects of steep-axis incision on corneal curvature in one-handed phacoemulsification. Int J Ophthalmol 12:1277-82. https://doi.org/10.18240/ijo.2019.08.07

85. Chen Y, Liao H, Sun Y, Shen X (2020) Short-term changes in the anterior segment and retina after small incision lenticule extraction. BMC Ophthalmol 20:397. https://doi.org/10.1186/s12886-020-01668-7

86. Deng MH, Chen S, Shi XF (2022) The binocular intraocular lens power difference in eyes with different axial lengths. Int J Ophthalmol 15:924-31. https://doi.org/10.18240/ijo.2022.06.09

87. Lin HS, Zhou MT, Li JX, Zheng XL, Ding YT, Ji YT, Wang XJ, Xie YQ, Liang YB (2024) Central anterior chamber depth correlated with white-to-white distance in normal, long, and short eyes. Int Ophthalmol 44:34. https://doi.org/10.1007/s10792-024-03014-y

88. Liu J, Wang Y, Huang W, Wang F, Xu Y, Xue Y, Wu M, Yu F, Gao R (2022) Comparison of the biometric parameters in patients with high myopia and anisometropia. BMC Ophthalmol 22:229. https://doi.org/10.1186/s12886-022-02450-7

89. Wang L, Liu S, Wang W, He M, Mo Z, Gong X, Xiong K, Li Y, Huang W (2021) Association between ocular biometrical parameters and diabetic retinopathy in Chinese adults with type 2 diabetes mellitus. Acta Ophthalmol 99:e661-e8. https://doi.org/10.1111/aos.14671

90. Janjetović Ž, Bušić M, Bosnar D, Barać J, Genda I (2019) Specific characteristics of ocular biometric factors in glaucomatous patients with pseudoexfoliative syndrome as measured by optical low-coherence reflectometry. Acta Clin Croat 58:87-94. https://doi.org/10.20471/acc.2019.58.01.11

91. Allam RSHM, Raafat KA, Elmohsen MNA (2022) Nasal trabeculo-ciliary angle and relative lens vault as predictors for intraocular pressure reduction following phacoemulsification. Eur J Ophthalmol 32:3019-28. https://doi.org/10.1177/11206721211055033

92. Fieß A, Marx‐Groß S, Wasielica‐Poslednik J, Nagler M, Schmidtmann I, Wild PS, Münzel T, Beutel ME, Lackner KJ, Pfeiffer N, Schuster AK (2022) Peripheral corneal thickness and associated factors – results from the population‐based German Gutenberg Health Study. Acta Ophthalmol 100:e1298-e305. https://doi.org/10.1111/aos.15057

93. Paritala A, Takkar B, Gaur N, Soni D, Ali MH, Rathi A (2022) Correlation of vitreous chamber depth with ocular biometry in high axial myopia. Indian J Ophthalmol 70:914-20. https://doi.org/10.4103/ijo.IJO_1201_21

94. Umesh Y, Saolapurkar K, Joshi P, Singh D (2023) Measurement of change in angle kappa and its correlation with ocular biometric parameters pre- and post-phacoemulsification. Indian J Ophthalmol 71:535–40. https://doi.org/10.4103/ijo.IJO_1641_22

95. Vagge A, Corazza P, Vagge R, Agosto G, Iester M, Camicione P, Ferro Desideri L, Traverso CE (2020) Biometric and refractive errors evaluation in patients with neurofibromatosis type 1. Eur J Ophthalmol 31:938-42. https://doi.org/10.1177/1120672120934402

96. Haider N, Parveen N, Rani S, Hussain Anwar S (2022) Effect of Pupil Dilation on Ocular Biometric Measurements in High Myopes. Pak J Ophthalmol 38:109-13. https://doi.org/10.36351/pjo.v38i2.1353

97. Kanclerz P, Przewłócka K, Wang X (2021) Inter-device measurement variability of vital data parameters for keratorefractive and cataract refractive surgery. Ther Adv Ophthalmol 13:1-6. https://doi.org/10.1177/25158414211045750

98. Hipólito-Fernandes D, Elisa Luís M, Maleita D, Gil P, Maduro V, Costa L, Marques N, Branco J, Alves N (2021) Intraocular lens power calculation formulas accuracy in combined phacovitrectomy: An 8-formulas comparison study. Int J Retina Vitreous 7:47. https://doi.org/10.1186/s40942-021-00315-7

99. Popov I, Waczulikova I, Stefanickova J, Valaskova J, Tomcikova D, Shiwani HA, Delev D, Rodrigo L, Saxena S, Kruzliak P, Krasnik V (2021) Analysis of biometric parameters of 2340 eyes measured with optical biometer Lenstar LS900 in a Caucasian population. Eur J Ophthalmol 32:213-20. https://doi.org/10.1177/1120672121998920

100. Monera Lucas CE, Escolano Serrano J, Tarazona Jaimes C, Romero Valero D, Moya Martínez A, Martínez Toldos JJ (2022) Repeatability and comparability of a new swept-source optical coherence tomographer in optical biometry. Arch Soc Esp Oftalmol (Engl Ed) 97:670-5. https://doi.org/10.1016/j.oftale.2022.04.003

101. Tasci YY, Yesilirmak N, Yuzbasioglu S, Ozdas D, Temel B (2021) Comparison of effects of mydriatic drops (1% cyclopentolate and 0.5% tropicamide) on anterior segment parameters. Indian J Ophthalmol 69:1802-7. https://doi.org/10.4103/ijo.IJO_2677_20

102. Altınel MG, Uslu H (2021) Agreement of keratometric readings measured using rotating Scheimpflug imaging, auto-refractokeratometer, and biograph in eyes with keratoconus. Int Ophthalmol 41:1659-69. https://doi.org/10.1007/s10792-021-01720-5

103. Cankaya C, Ozsoy E, Demirel EE, Polat N, Gunduz A (2020) Estimation of angle kappa and pupil barycentre configuration in myopic tilted disc syndrome. Clin Exp Optom 103:192-6. https://doi.org/10.1111/cxo.12897

104. Telek HH, Bilen RBA, Özdemir YA, Dinç DN, Çelikay O (2024) Comparison of keratometric values and anterior segment parameters measured using Scheimpflug Sirius topography and Lenstar biometry. Int Ophthalmol 44:103. https://doi.org/10.1007/s10792-024-03046-4

105. Zhang W, Pasricha ND, Kuo AN, Vann RR (2019) Influence of corneal diameter on surgically induced astigmatism in small-incision cataract surgery. Can J Ophthalmol 54:556-9. https://doi.org/10.1016/j.jcjo.2018.12.013

106. Lal B, Cantrell A, Ostrin LA (2024) Repeatability and agreement of the MYAH and Lenstar. Optom Vis Sci 101:157-63. https://doi.org/10.1097/OPX.0000000000002113

107. Placide J, Neves Da Silva HV, McCabe SE, Ronquillo YC, Moshirfar M (2021) Agreement of anterior segment measurements between four diagnostic imaging devices in myopic patients. Expert Rev Med Devices 18:1235-43. https://doi.org/10.1080/17434440.2021.2012153

108. Bruner C, Skanchy DF, Wooten JP, Chuang AZ, Kim G (2020) Anterior chamber lens sizing: Comparison of white-to-white and scleral spur-to-scleral spur methods. J Cataract Refract Surg 46:95-101. https://doi.org/10.1016/j.jcrs.2019.08.043

109. Liang T, Liu SN, Liu MG, Jiang ZT, Song S, Zhang AP (2022) Intraocular pressure fluctuation range and correction after small incision lenticule extraction. Int Eye Sci 22:14-21. https://doi.org/10.3980/j.issn.1672-5123.2022.1.03

110. Zeng H-B, Jiang J-D, Li R, Li J, Zeng W-J, Li X-K, Hu B, Zhou F, Yu H-Y, Xie J-G, Ning Z-N, Wang X (2023) The inflammatory cytokine profiles and ocular biometric characteristics of primary angle-closure glaucoma. J Int Med Res 51. https://doi.org/10.1177/03000605221147434

111. Zhou J, Gu W, Gao Y, Wang W, Zhang F (2022) Survival analysis of myopic regression after small incision lenticule extraction and femtosecond laser-assisted laser in situ keratomileusis for low to moderate myopia. Eye Vis (Lond) 9:28. https://doi.org/10.1186/s40662-022-00300-7

112. Xiong L, Wu J, Du H, Wang Z (2023) Comparison of formulas in the implantable collamer lens vault prediction. Medicine (Baltimore) 102:e36542. https://doi.org/10.1097/MD.0000000000036542

113. Xu G, Wu G, Du Z, Zhu S, Guo Y, Yu H, Hu Y (2021) Distribution of white-to-white corneal diameter and anterior chamber depth in chinese myopic patients. Front Med 8:732719. https://doi.org/10.3389/fmed.2021.732719

114. Tang C, Sun T, Duan H, Liu Y, Qi H (2023) Evaluation of the performance of two nomograms and four vault prediction formulas for implantable collamer lens size selection. J Refract Surg 39:456-61. https://doi.org/10.3928/1081597X-20230605-01

115. Lin Q, Shen Z (2022) Effect of white-to-white corneal diameter on biomechanical indices assessed by Pentacam Scheimpflug corneal tomography and corneal visualization Scheimpflug technology. Int Ophthalmol 42:1537-43. https://doi.org/10.1007/s10792-021-02144-x

116. Rateaux M, Bremond-Gignac D, Robert MP (2022) From monocular photograph to angle lambda: A new clinical approach for quantitative assessment. J Binocul Vis Ocul Motil 72:169-75. https://doi.org/10.1080/2576117X.2022.2083541

117. Abdi P, Farsiani AR, Fallah Tafti MR, Latifi G, Abdi P (2023) Effect of ocular biometric factors on corneal biomechanical properties. Int Ophthalmol 43:1877-88. https://doi.org/10.1007/s10792-022-02587-w

118. Fairaq R, Almutlak M, Almazyad E, Badawi AH, Ahad MA (2021) Outcomes and complications of implantable collamer lens for mild to advance keratoconus. Int Ophthalmol 41:2609-18. https://doi.org/10.1007/s10792-021-01820-2

119. Kim DR, Yoon YC, Whang W-J, Hwang HS, Na K-S (2024) Ocular parameters associated with visual performance of enhanced monofocal intraocular lens. BMC Ophthalmol 24:74. https://doi.org/10.1186/s12886-024-03316-w

120. Lin Q, Yang D, Zhou X (2022) Early outcomes of anterior segment parameters after implantable collamer lens V4c implantation. BMC Ophthalmol 22:429. https://doi.org/10.1186/s12886-022-02656-9

121. Yang F, Bao Y (2023) Correlations of corneal curvature with corneal spherical aberration and anterior chamber parameters in eyes with shallow Anterior chambers. Ophthalmol Ther 12:239-49. https://doi.org/10.1007/s40123-022-00608-6

122. Liang G, Kai J-Y, Li D-L, Yin Z-J, Li Y-Z, Ma R, Zheng Y-J, Qin Y, Pan C-W (2024) Distribution and determinants of corneal volume among healthy young Chinese adults: A cross-sectional study. BMC Ophthalmol 24:59. https://doi.org/10.1186/s12886-024-03342-8

123. Xu K, Liu X, Lei Y, Qi H, Zhang C (2021) Use of neural networks to predict vault values after implantable collamer lens surgery. Graefes Arch Clin Exp Ophthalmol 259:3795-803. https://doi.org/10.1007/s00417-021-05294-x

124. Ma Y, Ma Y, Feng C, Shen M, Yuan Y (2020) Ocular biometric parameters are associated with non-contact tonometry measured intraocular pressure in non-pathologic myopic patients. Int Ophthalmol 40:431-7. https://doi.org/10.1007/s10792-019-01203-8

125. Paulo JD, Hurtado LS, Donado-Gómez JH, Lopera NG (2021) Characterization of ocular biometric parameters in colombian candidates for cataract surgery. Rev Fac Med Univ Nac Auton Mex 69:e78870. https://doi.org/10.15446/revfacmed.v69n2.78870

126. Natung T, Shullai W, Nongrum B, Thangkhiew L, Baruah P, Phiamphu ML (2019) Ocular biometry characteristics and corneal astigmatisms in cataract surgery candidates at a tertiary care center in North-East India. Indian J Ophthalmol 67:1417-23. https://doi.org/10.4103/ijo.IJO_1353_18

127. Nakao S-y, Miyake M, Hosoda Y, Nakano E, Mori Y, Takahashi A, Ooto S, Tamura H, Tabara Y, Yamashiro K, Matsuda F, Tsujikawa A, Nakayama T, Sekine A, Kosugi S (2021) Myopia prevalence and ocular biometry features in a Ggeneral japanese population: The nagahama study. Ophthalmology 128:522-31. https://doi.org/10.1016/j.ophtha.2020.08.023

128. Loh UL, Qamarruddin FA, Hussein A (2022) The study of relationship between ocular biometry and exophthalmometry in adult Malay population of Kelantan, Malaysia. Taiwan J Ophthalmol 12:44-52. https://doi.org/10.4103/tjo.tjo_88_20

129. Almorín-Fernández-Vigo I, Sánchez-Guillén I, Fernández-Vigo JI, Macarro-Merino A, Kudsieh B, Fernández-Vigo C, Fernández-Vigo JA (2019) Normative Pentacam anterior and posterior corneal elevation measurements: Effects of age, sex, axial length and white-to-white. Int Ophthalmol 39:1955-63. https://doi.org/10.1007/s10792-018-1028-6

130. Dikmetas O, Deliktas O, Toprak H, Karahan S, Kocabeyoglu S, Cankaya AB (2021) Correlation of ocular biometric parameters and macular ganglion cell layer in normal eyes. Semin Ophthalmol 36:812-7. https://doi.org/10.1080/08820538.2021.1922711

131. Vingopoulos F, Nair A, See CW, Iyengar N, Haberman I, Sperber L, Lazzaro DR, Singh R, Ho A, Gupta O, Sharma S, Modi Y (2021) Position of in-the-bag posterior chamber intraocular lenses relative to the limbus: Applications to scleral-sutured lenses. Retina 41:1533-40. https://doi.org/10.1097/IAE.0000000000003044

132. Tu R, Yu J, Savini G, Ye J, Ning R, Xiong J, Chen S, Huang J (2020) Agreement between two optical biometers based on large coherence length SS-OCT and Scheimpflug imaging/partial coherence interferometry. J Refract Surg 36:459-65. https://doi.org/10.3928/1081597X-20200420-02

133. Chen S, Zhang Q, Savini G, Zhang S, Huang X, Yu J, Wang Y, Ning R, Huang J, Tu R (2022) Comparison of a new optical biometer that combines Scheimpflug imaging with partial coherence interferometry with that of an optical biometer based on swept-source optical coherence tomography and Placido-disk topography. Front Med 8:814519. https://doi.org/10.3389/fmed.2021.814519

134. Jiang Y, Chen X, Cheng M, Li B, Lei Y, Lin IC, Xu G, Mingwei L, Zhou X, Wang X (2024) Immediate versus delayed sequential bilateral ICL implantation: A retrospective comparison of vault height and visual outcomes. Asia Pac J Ophthalmol (Phila) 13:100075. https://doi.org/10.1016/j.apjo.2024.100075

135. Chen X, Shen Y, Jiang Y, Cheng M, Lei Y, Li B, Niu L, Chen J, Wang X, Zhou X (2023) Predicting vault and size of posterior chamber phakic intraocular lens using sulcus to sulcus-optimized artificial intelligence technology. Am J Ophthalmol 255:87-97. https://doi.org/10.1016/j.ajo.2023.06.024

136. Sardari S, Fotouhi A, Jafarzadehpur E, Khabazkhoob M (2023) Agreements’ profile of Scheimpflug-based optical biometer with gold standard partial coherence interferometry. Int J Ophthalmol 16:2095-104. https://doi.org/10.18240/ijo.2023.12.24

137. Kanclerz P, Hoffer KJ, Bazylczyk N, Wang X, Savini G (2023) Optical biometry and IOL calculation in a commercially available optical coherence tomography device and comparison with Pentacam AXL. Am J Ophthalmol 246:236-41. https://doi.org/10.1016/j.ajo.2022.09.022

138. Muzyka-Woźniak M, Oleszko A (2019) Comparison of anterior segment parameters and axial length measurements performed on a Scheimpflug device with biometry function and a reference optical biometer. Int Ophthalmol 39:1115-22. https://doi.org/10.1007/s10792-018-0927-x

139. Supiyaphun C, Rattanasiri S, Jongkhajornpong P (2020) Comparison of anterior segment parameters and axial length using two Scheimpflug devices with integrated optical biometers. Clin Ophthalmol 14:3487-94. https://doi.org/10.2147/OPTH.S278701

140. Moshirfar M, Tenney S, McCabe S, Schmid G (2022) Repeatability and reproducibility of the Galilei G6 and its agreement with the Pentacam® AXL in optical biometry and corneal tomography. Expert Rev Med Devices 19:375-83. https://doi.org/10.1080/17434440.2022.2075725

141. Du GP, Guo HL, Jiang JJ, Wang LQ (2021) Clinical efficacy of Toric ICL implantation and its effect on corneal astigmatism and aberration. Int Eye Sci 21:1675-9. https://doi.org/10.3980/j.issn.1672-5123.2021.10.02

142. Khan MA, Tan Q, Sun W, Cai W, Zhao L, Lin D (2022) Prediction of excessively low vault after implantable collamer lens implantation using iris morphology. Front Med 9:1029350. https://doi.org/10.3389/fmed.2022.1029350

143. Ning J, Sun S, Zhang Q, Jin L, Liu X, Xu J, Zhang L (2024) Corneal densitometry in Chinese adults with healthy corneas: associations with sex, age, ocular metrics, and optical characteristics. BMC Ophthalmol 24:230. https://doi.org/10.1186/s12886-024-03500-y

144. Miao A, Tang Y, Zhu X, Qian D, Zheng T, Lu Y (2022) Associations between anterior segment biometry and high axial myopia in 3438 cataractous eyes in the Chinese population. BMC Ophthalmol 22:71. https://doi.org/10.1186/s12886-022-02300-6

145. Ye Y, Xu Y, Zhang Z, Niu L, Shi W, Wang X, Zhou X, Zhao J (2024) The long-term visual quality and rotational stability after ICL/TICL V4c Iimplantation in individuals with high myopia older than 40 years. J Refract Surg 40:e381-e91. https://doi.org/10.3928/1081597X-20240422-04

146. Ding L, Wang J, Niu L, Shi W, Qian Y (2020) Pentacamscheimpflug tomography findings in Chinese patients with different corneal diameters. J Refract Surg 36:688-95. https://doi.org/10.3928/1081597X-20200730-02

147. Xiong J, Xu J, Zhou M, Liu J, Wang Q, Yin X, Deng Y, Luo X, Wang N, Gui F, Yu K, Liu J, Zhu Z, Cheng C, Yu Y (2024) Mesopic pupil indices as potential risk factors for glare disability after intraocular implantable collamer lens implantation: prospective study. J Cataract Refract Surg 50:565-71. https://doi.org/10.1097/j.jcrs.0000000000001420

148. Liu J, Wang Y, Zou H, Li M (2021) Influence of corneal shape parameters on corneal deformation responses measured with a Scheimpflug camera. Int Ophthalmol 41:2853-9. https://doi.org/10.1007/s10792-021-01844-8

149. Yiming Y, Xi C, Huan Y, Liming C, Na Y, Pei C, Ying Y, Yan L, Keming Y (2023) Evaluation of ciliary body morphology and position of the implantable collamer lens in low-vault eyes using ultrasound biomicroscopy. J Cataract Refract Surg 49:1133-9. https://doi.org/10.1097/j.jcrs.0000000000001285

150. Yu N, Hou X, Luo Y, Chen X, Zhuang J, Yu K (2023) Comparison of refractive outcomes and optical zone decentration after SMILE between patients with symmetrical and asymmetrical high astigmatism. J Refract Surg 39:273-80. https://doi.org/10.3928/1081597X-20230207-01

151. Gharieb HM, Shalaby HS, Othman IS (2020) Repeatability and interchangeability of topometric, anterior chamber and corneal wavefront data between two Scheimpflug camera devices. Clin Ophthalmol 14:3801-10. https://doi.org/10.2147/OPTH.S274303

152. Bandlitz S, Nakhoul M, Kotliar K (2022) Daily variations of corneal white-to-white diameter measured with different methods. Clin Optom (Auckl) 14:173-81. https://doi.org/10.2147/OPTO.S360651

153. Herber R, Lenk J, Pillunat LE, Raiskup F (2022) Agreement and repeatability of corneal tomography in healthy eyes using a new swept-source OCT, a rotating Scheimpflug camera, and a dual Scheimpflug–Placido system. J Cataract Refract Surg 48:190-8. https://doi.org/10.1097/j.jcrs.0000000000000734

154. Karmiris E, Tsiogka A, Tsiripidis K, Papakonstantinou E, Georgalas I, Chalkiadaki E (2024) Correlations of corneal endothelial morphology and corneal thickness withanterior segment parameters in healthy individuals. Cornea 43:764-70. https://doi.org/10.1097/ICO.0000000000003515

155. Momeni-Moghaddam H, Maddah N, Wolffsohn JS, Etezad-Razavi M, Zarei-Ghanavati S, Akhavan Rezayat A, Moshirfar M (2019) The effect of cycloplegia on the ocular biometric and anterior segment parameters: A cross-sectional study. Ophthalmol Ther 8:387-95. https://doi.org/10.1007/s40123-019-0187-5

156. Alshehri O, Abdelaal AM, Abudawood G, Hijazi H, Alqassimi A, Khan MA, Alsharif S (2022) Normative values for corneal tomography and comparison of both eyes in young saudimales with 20/20 vision using Pentacam-HR Scheimpflug imaging. Clin Ophthalmol 16:2631-7. https://doi.org/10.2147/OPTH.S376411

157. Cerpa Manito S, Sánchez Trancón A, Torrado Sierra O, Baptista AM, Serra PM (2021) Biometric and ICL-related risk factors associated to sub-optimal vaults in eyes implanted with implantable collamer lenses. Eye Vis (Lond) 8:26. https://doi.org/10.1186/s40662-021-00250-6

158. Beltrán-Murcia J, Capelo LÁ-R, Blázquez-Sánchez V (2023) Analysis of vault prediction in phakic implantable phakic collamer lenses: Manufacturer’s calculator vs theoretical formulae vs clinical practice. Graefes Arch Clin Exp Ophthalmol 261:2403-9. https://doi.org/10.1007/s00417-023-06016-1

159. Seguí-Crespo M, Ariza-Gracia MÁ, Sixpene NdLD, Piñero DP (2019) Geometrical characterization of the corneo-scleral transition in normal patients with Fourier domain optical coherence tomography. Int Ophthalmol 39:2603-9. https://doi.org/10.1007/s10792-019-01109-5

160. Llorens-Quintana C, Li Y, Chen S, Fujimoto JG, Huang D (2023) Characterization of the external limbus on corneoscleral topography with ultrawide-field optical coherence tomography. Cont Lens Anterior Eye 46:102065. https://doi.org/10.1016/j.clae.2023.102065

161. Boyd BM, Bai J, Borgstrom M, Belin MW (2020) Comparison of Chinese and North American tomographic parameters and the implications for refractive surgery screening. Asia Pac J Ophthalmol (Phila) 9:117-25. https://doi.org/10.1097/APO.0000000000000273

162. Guo XX, You R, Li SS, Yang XF, Zhao L, Zhang F, Wang YL, Chen X (2019) Comparison of ocular parameters of two biometric measurement devices in highly myopic eyes. Int J Ophthalmol 12:1548-54. https://doi.org/10.18240/ijo.2019.10.05

163. Yuan J, Wu S, Hu Z, Chen C, Ye S, Ye J (2024) Clinical observation of posterior-chamber phakic implantable collamer lens V4c implantation in myopic patients with shallow anterior chamber depth: A retrospective, consecutive observational study. J Ophthalmol 2024:3181569. https://doi.org/10.1155/2024/3181569

164. Ma Y, Li Q, Dong Y, Yi X (2024) Unexpected findings: Loss of corneal endothelial cells in Uygur patients with exfoliation syndrome. Int Ophthalmol 44:71. https://doi.org/10.1007/s10792-024-02913-4

165. Xu J, Lin P, Zhang S, Lu Y, Zheng T (2022) Risk factors associated with intraocular lens decentration after cataract surgery. Am J Ophthalmol 242:88-95. https://doi.org/10.1016/j.ajo.2022.05.005

166. Chen X, Han T, Zhao W, Wang X, Xu Y, Cheng M, Wang X, Zhou X (2021) Effect of the difference between the white-to-white and sulcus-to-sulcus on vault and the related factors after ICL implantation. Ophthalmol Ther 10:947-55. https://doi.org/10.1007/s40123-021-00386-7

167. Fan W, Zhang C, Ge L, Su N, Chen J, Song S, Wang Y, Yuan S (2024) Prediction model for elevated intraocular pressure risk after silicone oil filling based on clinical features. Front Med 10:1340198-. https://doi.org/10.3389/fmed.2023.1340198

168. Sun J, Bai H, Cui W, Wu X (2024) Comparison of clinical outcome after implantation of two toric intraocular lenses with different haptic type: a prospective randomized controlled trial. Graefes Arch Clin Exp Ophthalmol 262:847-55. https://doi.org/10.1007/s00417-023-06232-9

169. Zhang W, Li F, Li L, Zhang J (2023) A quantitative study of the effect of ICL orientation selection on post-operative vault and model-assisted vault prediction. Front Neurol 14:1136579. https://doi.org/10.3389/fneur.2023.1136579

170. Elkitkat RS, Fouad YA, Shams A, Hamza I (2020) Normative values of corneal spherical aberration, pupil size, and other key refractive and topographic parameters in a large cohort of egyptian cataract surgery candidates. Clin Ophthalmol 14:4571-7. https://doi.org/10.2147/OPTH.S288738

171. Debellemanière G, Mechleb N, Bernier T, Ancel J-M, Gauvin M, Wallerstein A, Saad A, Gatinel D (2024) The development of a thick-lens post-myopic laser vision correction intraocular lens calculation formula. Am J Ophthalmol 262:40-7. https://doi.org/10.1016/j.ajo.2023.09.023

172. Juliane M, Anne-Isabel L, Myriam C, Vasyl D, Toam K, Linke SJ (2022) Biometry and intraocular lens power calculation by combined Scheimpflug-Placido disc versus optical interferometry devices. J Ophthalmic Vis Res 17:453-61. https://doi.org/10.18502/jovr.v17i4.12349

173. Szalai E, Csutak A (2022) Comparative analysis of two optical biometry devices: high wavelength swept source OCT versus partial coherence interferometry. Int Ophthalmol 42:627-34. https://doi.org/10.1007/s10792-021-02036-0

174. Singh K, Gupta S, Moulick PS, Bhargava N, Sati A, Kaur G (2019) Study of distribution of white-to-white corneal diameter and anterior chamber depth in study population obtained with optical biometry using intraocular lens (IOL) master. Med J Armed Forces India 75:400-5. https://doi.org/10.1016/j.mjafi.2018.06.001

175. Almorín-Fernández-Vigo I, Sánchez-Guillén I, Fernández-Vigo JI, Burgos-Blasco B, De-Pablo-Gómez-de-Liaño L, Fernández-Vigo JÁ, Macarro-Merino A (2023) Normative topographic anterior and posterior corneal astigmatism: Axis distribution and its relations with ocular and biometric parameters. J Clin Med 12:3664. https://doi.org/10.3390/jcm12113664

176. Moshirfar M, Han KD, Jaafar MA, Santos JM, Theis JS, Stoakes IM, Hoopes PC (2024) Comparative evaluation of multiple nomograms for predicting postoperative vault after implantable collamer lens surgery. J Cataract Refract Surg 50:64-71. https://doi.org/10.1097/j.jcrs.0000000000001304

177. Almorín-Fernández-Vigo I, Sánchez-Guillén I, Fernández-Vigo JI, De-Pablo-Gómez-de-Liaño L, Kudsieh B, Fernández-Vigo JÁ, Macarro-Merino A (2021) Agreement between optical coherence and Scheimpflug tomography: Vault measurements and reproducibility after implantable collamer lens implantation. J Fr Ophtalmol 44:1370-80. https://doi.org/10.1016/j.jfo.2021.03.007

178. Teixeira EGRM, Gomes BF, Santana JD, Santhiago MR, Costa AA, Moraes HV (2023) Agreement between corneal diameter measurements obtained with an optical biometer and a Placido-based topographer. Arq Bras Oftalmol 86:e2021-0325. https://doi.org/10.5935/0004-2749.2021-0325

179. Dong J, Yao J, Chang S, Kanclerz P, Khoramnia R, Wang X (2023) Evaluation of ocular diameter parameters using swept-source optical coherence tomography. Medicina (Kaunas) 59:899. https://doi.org/10.3390/medicina59050899

180. Panthier C, Rouger H, Gozlan Y, Moran S, Gatinel D (2022) Comparative analysis of 2 biometers using swept-source OCT technology. J Cataract Refract Surg 48:26-31. https://doi.org/10.1097/j.jcrs.0000000000000704

181. Hasan SM, Theilig T, Lehmann T, Meller D (2023) Factors correlated with mid-term morphology of functional blebs following implantation of preserflo MicroShunt using AS-OCT. Transl Vis Sci Technol 12:4. https://doi.org/10.1167/tvst.12.11.4

182. Sorkin N, Achiron A, Abumanhal M, Abulafia A, Cohen E, Gutfreund S, Mandelblum J, Varssano D, Levinger E (2022) Comparison of two new integrated SS-OCT tomography and biometry devices. J Cataract Refract Surg 48:1277-84. https://doi.org/10.1097/j.jcrs.0000000000000974

183. Kim T, Kim SJ, Lee BY, Cho HJ, Sa BG, Ryu IH, Kim JK, Lee IS, Han E, Kim H, Yoo TK (2023) Development of an implantable collamer lens sizing model: A retrospective study using ANTERION swept-source optical coherence tomography and a literature review. BMC Ophthalmol 23:59. https://doi.org/10.1186/s12886-023-02814-7

184. Yu NH, Kang KH, Tchah H, Koh K (2024) A comparative study of two phakic posterior chamber implantable lenses. Medicine (Baltimore) 103:e38194. https://doi.org/10.1097/MD.0000000000038194

185. Escolano Serrano J, Tarazona Jaimes CP, Monera Lucas CE, Romero Valero D, Moya Martínez A, Martínez Toldos JJ (2022) Intraobserver repeatability of tomographic, pachymetric, and anatomical measurements in healthy eyes using a new swept-source optical coherence topographer. Cornea 41:589-603. https://doi.org/10.1097/ICO.0000000000002799

186. Montés-Micó R, Tañá-Rivero P, Aguilar-Córcoles S, Ruíz-Mesa R (2020) Assessment of anterior segment measurements using a high-resolution imaging device. Expert Rev Med Devices 17:969-79. https://doi.org/10.1080/17434440.2020.1816463

187. Tañá-Rivero P, Pastor-Pascual F, Crespo M, Rodríguez-Prats JL, Muñoz-Tomás JJ, Montés-Micó R (2020) Posterior-chamber phakic intraocular lens implantation in patients over 40 years of age. J Ophthalmol 2020:7457902. https://doi.org/10.1155/2020/7457902

188. Abicca I, Schiano-Lomoriello D, Gilardi M, Giannini D, Dinu V, Coutinho C, Savini G (2024) Repeatability of automatic measurements by an anterior segment swept-source OCT biometer in patients with keratoconus. J Refract Surg 40:e445-e52. https://doi.org/10.3928/1081597X-20240514-03

189. Namkung S, Boyle AB, Li Y, Gokul A, McGhee C, Ziaei M (2022) Repeatability and agreement of horizontal corneal diameter measurements between scanning-slit topography, dual rotating Scheimpflug camera with Placido disc tomography, Placido disc topography, and optical coherence tomography. Cornea 41:1392-7. https://doi.org/10.1097/ICO.0000000000002964

190. Chen J, Zhang Y, Huang Y, Ng TK, Huang C (2023) Relatively anterior lens position in primary angle-closure glaucoma eyes with long axial length. Indian J Ophthalmol 71:1941-7. https://doi.org/10.4103/ijo.IJO_1890_22

191. Cui W, Wu X, Ren Q, Liu K, Kong F, Wu J (2023) A new formula based on new parameters for predicting postoperative vault after posterior chamber intraocular lens implantation: A retrospective study. Quant Imaging Med Surg 13:5502-10. https://doi.org/10.21037/qims-22-1425

192. Li Y, Zou Z, Xu S, Yu J, Ye Q, Li K, Xiao Y, Savini G, Schiano-Lomoriello D, Zhou X, Yao M, Huang J (2023) Evaluation of a new all-in-one optical biometer and comparison with a validated swept-source OCT biometer. J Refract Surg 39:825-30. https://doi.org/10.3928/1081597X-20231018-02

193. Jamali A, Naghdi T, Abardeh MH, Jamalzehi M, Khalajzadeh M, Kamangar M, Tehranchi N, Nabovati P (2021) Ocular biometry characteristics in cataract surgery candidates: A cross-sectional study. Med Hypothesis Discov Innov Ophthalmol 10:11-7. https://doi.org/10.51329/mehdiophthal1416

194. Gharieb Ibrahim HM, Gharieb HM, Othman IS (2022) Angle κ measurement and its correlation with other ocular parameters in normal population by a new imaging modality. Optom Vis Sci 99:580-8. https://doi.org/10.1097/OPX.0000000000001910

195. Nemeth G, Modis L, Jr. (2019) Ocular measurements of a swept-source biometer: Repeatability data and comparison with an optical low-coherence interferometry biometer. J Cataract Refract Surg 45:789-97. https://doi.org/10.1016/j.jcrs.2018.12.018

196. Matsumoto Y, Azuma Y, Karasawa Y, Suzuki N (2024) Implantation of hydrophobic acrylic toric intraocular lens with high-water contents using swept-source optical coherence tomography biometer integrated with a surgical guiding system. Clin Ophthalmol 18:1117-24. https://doi.org/10.2147/OPTH.S456609

197. Shammas HJ, Yu F, Shammas MC, Jivrajka R, Hakimeh C (2022) Predicted vs measured posterior corneal astigmatism for toric intraocular lens calculations. J Cataract Refract Surg 48:690-6. https://doi.org/10.1097/j.jcrs.0000000000000819

198. Shammas HJ, Taroni L, Pellegrini M, Shammas MC, Jivrajka RV (2022) Accuracy of newer intraocular lens power formulas in short and long eyes using sum-of-segments biometry. J Cataract Refract Surg 48:1113-20. https://doi.org/10.1097/j.jcrs.0000000000000958

199. Wu H, Luo D-q, Chen J, Wang H, Zhong D-j (2024) Comparison of the accuracy of seven vault prediction formulae for implantable collamer lens implantation. Ophthalmol Ther 13:237-49. https://doi.org/10.1007/s40123-023-00844-4

200. Wu H, Zhong DJ, Luo DQ, Zhang LY, Liu J, Wang H (2023) Improvement in the ideal range of vault after implantable collamer lens implantation: A new vault prediction formula. Front Med 10:1132102-. https://doi.org/10.3389/fmed.2023.1132102

201. Hamza MN, Roshdy MM, Seleet MM, Raggal TME (2021) Correlation between ocular biometric parameters and corneal endothelium in a sample of young Egyptian adults. Med Hypothesis Discov Innov Ophthalmol 10:121-8. https://doi.org/10.51329/MEHDIOPHTHAL1430

202. Tuncer I, Zengin MÖ, Yıldız S (2021) The effect of cycloplegia on the ocular biometry and intraocular lens power based on age. Eye 35:676-81. https://doi.org/10.1038/s41433-020-01131-3

203. Yaşa D, Köse B, Ağca A (2020) Rotational stability of a new posterior chamber toric phakic intraocular lens. J Ophthalmol 2020:1624632. https://doi.org/10.1155/2020/1624632

204. Fukumitsu H, Camps VJ, Miraflores S, Piñero DP (2021) Relationship between medium-term changes in intraocular lens position and refraction after cataract surgery with two different models of monofocal lenses. J Clin Med 10:3856. https://doi.org/10.3390/jcm10173856

205. Han ES, Kim M (2019) Evaluation of biometry and corneal astigmatism in cataract surgery patients in Northern United Arab Emirates. Int Ophthalmol 39:2807-13. https://doi.org/10.1007/s10792-019-01127-3

206. McLintock C, Niyazmand H, Seo S, Barrett G, Kumar Nilagiri V, Karimian S, McKelvie J (2022) Agreement between a new swept-source ocular coherence tomography and a Placido disc-dual Scheimpflug ocular biometric devices. Eur J Ophthalmol 33:905-11. https://doi.org/10.1177/11206721221143160

207. Hashemi H, Miraftab M, Panahi P, Asgari S (2022) Biometry and intraocular power calculation using a swept-source optical coherence tomography: A repeatability and agreement study. Indian J Ophthalmol 70:2845-50. https://doi.org/10.4103/ijo.IJO_249_22

208. Zheng Q, Hu M, Li ZL, Chang PJ, Zhao YE (2021) Assessment of anterior chamber angle changes after phacoemulsification with swept-source OCT. Int J Ophthalmol 14:1527-32. https://doi.org/10.18240/ijo.2021.10.08

209. Jadidi K, Mosavi SA, Nejat F, Mohammadi N, Aghamolaei H, Daryabari S-H, Torabi H, Alishiri A (2019) Use of low-vault posterior chamber collagen copolymer phakic intraocular lenses for the correction of myopia: A 3-year follow-up. Graefes Arch Clin Exp Ophthalmol 257:1555-60. https://doi.org/10.1007/s00417-019-04336-9

210. Nakamura T, Isogai N, Kojima T, Yoshida Y, Sugiyama Y (2020) Optimization of implantable collamer lens sizing based on swept-source anterior segment optical coherence tomography. J Cataract Refract Surg 46:742-8. https://doi.org/10.1097/j.jcrs.0000000000000134

211. Igarashi A, Shimizu K, Kato S (2021) Assessment of the vault after implantable collamer lens implantation using the KS formula. J Refract Surg 37:636-41. https://doi.org/10.3928/1081597X-20210610-06

212. Akaishi M, Teshigawara T, Hata S, Meguro A, Mizuki N (2023) Multiple linear regression model for improving accuracy of capsulorhexis size calculation in femtosecond laser-assisted cataract surgery for adults: A retrospective single-center study. BMC Ophthalmol 23:19. https://doi.org/10.1186/s12886-023-02776-w

213. Igarashi A, Shimizu K, Kato S, Kamiya K (2019) Predictability of the vault after posterior chamber phakic intraocular lens implantation using anterior segment optical coherence tomography. J Cataract Refract Surg 45:1099-104. https://doi.org/10.1016/j.jcrs.2019.02.020

214. Sorkin N, Zadok T, Barrett GD, Chasid O, Abulafia A (2023) Comparison of biometry measurements and intraocular lens power prediction between 2 SS‐OCT–based biometers. J Cataract Refract Surg 49:460-6. https://doi.org/10.1097/j.jcrs.0000000000001146

215. Venkataraman AP, Domínguez-Vicent A, Selin P, Brautaset R, Montés-Micó R (2024) Precision of a new SS-OCT biometer to measure anterior segment parameters and agreement with 3 instruments with different measurement principles. J Cataract Refract Surg 50:486-91. https://doi.org/10.1097/j.jcrs.0000000000001380

216. Li C, Zhang J, Yin X, Li J, Cao Y, Lu P (2019) Distribution and related factors of corneal regularity and posterior corneal astigmatism in cataract patients. Clin Ophthalmol 13:1341-52. https://doi.org/10.2147/OPTH.S212946

217. Khorrami-Nejad M, Khodaparast M, Abdulkadhim IA, Azizi E, Rashidi F, Damanpak V, Hashemian H (2024) A comparison of Scansys and Sirius tomography in healthy eyes. BMC Ophthalmol 24:138. https://doi.org/10.1186/s12886-024-03389-7

218. Kilic D, Akmaz B, Akay F, Guven YZ, Oruk GG (2021) Changes in anterior segment parameters and presence of dry eye disease in patients with acromegaly: A Sirius topography study combined with meibography. Growth Horm IGF Res 60-61:101424. https://doi.org/10.1016/j.ghir.2021.101424

219. Kim BK, Chung YT (2021) Clinical results of Visian implantable collamer lens implantation according to various sizes and implantation angles. Eur J Ophthalmol 32:2041-50. https://doi.org/10.1177/11206721211033468

220. Zhou Ta, Jiang H, Wang Y, Xie C, Xia J, Shen Y (2020) Comparison of anterior chamber angle changes following phakic intraocular lens with and without a central hole implantation for moderate to high myopic eyes. Medicine (Baltimore) 99:e23434. https://doi.org/10.1097/MD.0000000000023434

221. Gonzalez-Lopez F, Bilbao-Calabuig R, Mompean B, Luezas J, Ortega-Usobiaga J, Druchkiv V (2019) Determining the potential role of crystalline lens rise in vaulting in posterior chamber phakic collamer lens surgery for correction of myopia. J Refract Surg 35:177-83. https://doi.org/10.3928/1081597X-20190204-01

222. Wang Y, Wan T, Liu L, Xue Y, Chen X, Savini G, Schiano-Lomoriello D, Zhou X, Yu J, Huang J (2023) Agreement between a new optical low coherence reflectometry biometer and an anterior segment optical coherence tomographer. Eye Vis (Lond) 10:13. https://doi.org/10.1186/s40662-023-00330-9

223. Wang H, Zhu L-S, Pang C-J, Fan Q (2024) Repeatability assessment of anterior segment measurements in myopic patients using an anterior segment OCT with Placido corneal topography and agreement with a swept-source OCT. BMC Ophthalmol 24:182. https://doi.org/10.1186/s12886-024-03448-z

224. Gessesse GW, Debela AS, Anbesse DH (2020) Ocular biometry and their correlations with ocular and anthropometric measurements among Ethiopian adults. Clin Ophthalmol 14:3363-9. https://doi.org/10.2147/OPTH.S277359

225. Yu J, Zhao G, Lei CS, Wan T, Ning R, Xing W, Ma X, Pan H, Savini G, Schiano-Lomoriello D, Zhou X, Huang J (2024) Repeatability and reproducibility of a new fully automatic measurement optical low coherence reflectometry biometer and agreement with swept-source optical coherence tomography-based biometer. Br J Ophthalmol 108:673-8. https://doi.org/10.1136/bjo-2023-323268

226. Yoo Y-S, Whang W-J, Kim H-S, Joo C-K, Yoon G (2019) Preoperative biometric measurements with anterior segment optical coherence tomography and prediction of postoperative intraocular lens position. Medicine (Baltimore) 98:e18026. https://doi.org/10.1097/MD.0000000000018026

227. Reinstein DZ, Vida RS, Archer TJ (2021) Visual outcomes, footplate position and vault achieved with the Visian implantable collamer lens for myopic astigmatism. Clin Ophthalmol 15:4485-97. https://doi.org/10.2147/OPTH.S330879

228. Sella R, Bu JJ, Lian RR, Hu JQ, Gali HE, Walker EH, Livny E, Afshari NA (2024) Axial length and pharmacologic pupillary dilation in highly myopic patients. Graefes Arch Clin Exp Ophthalmol 262:1531-8. https://doi.org/10.1007/s00417-023-06296-7

229. Yu J, Lin X, Huang X, Xu Z, Ning R, Li K, Savini G, Schiano-Lomoriello D, Zhou X, Huang J (2024) Evaluation of a new dynamic real-time visualization 25 kHz swept-source optical coherence tomography based biometer. Eye Vis (Lond) 11:9. https://doi.org/10.1186/s40662-024-00377-2

230. Zhang T, Huang F, Gao N, Du M, Cheng H, Huang W, Ji Y, Zheng S, Wan W, Hu K (2023) Three-dimensional quantitative description of the implantable collamer lens in the ocular anterior segment of patients with myopia. Am J Ophthalmol 252:59-68. https://doi.org/10.1016/j.ajo.2023.03.005

Title: Mean Human Corneal Diameter and Palpebral Fissure Lengths as Scales for Forensic Analysis of Photographed Faces: An Analytical Review

Journal Name: International Journal of Legal Medicine

Author Names: Sean S. Healy & Carl N. Stephan

Affiliation: Laboratory for Human Craniofacial and Skeletal Identification (HuCS-ID Lab), School of Biomedical Sciences, The University of Queensland, Brisbane, Australia, 4072.

Corresponding Author Email: sean.healy@uq.net.au
