# Supplementary material for: Mean human corneal diameter and palpebral fissure lengths as scales for forensic analysis of photographed faces: an analytical review*
Source: Int J Legal Med. 2026 Feb 23;140(3):1529–46. doi: 10.1007/s00414-026-03733-0 (PMC13161299; doi:10.1007/s00414-026-03733-0)
Supplement: Supplementary file 4 — Supplementary Material 4 [file 414_2026_3733_MOESM4_ESM.docx]

**Supplementary Material 4**

**Weighted Mean and Combined Standard Deviation Equations**

**Equation 1**. Weighted Mean

$$\bar{x}= \frac{\sum_{i=1}^{n} N_{i}x_{i}}{\sum_{i=1}^{n} N_{i}}$$

Where,

N = Number of observations within sample
x = Mean of sample

**Equation 2**. Combined Standard Deviation

$$\sigma_{combined}=\sqrt{\frac{\sum_{i=1}^{n} N_{i}\sigma_{i}^{2}+N_{i}{(x_{i}-\bar{x})}^{2}}{\sum_{i=1}^{n} N_{i}}}$$

Where,

N = Number of observations within sample
σ = Standard deviation of sample

x = Mean of sample

x̄ = Weighted mean of total sample

Title: Mean Human Corneal Diameter and Palpebral Fissure Lengths as Scales for Forensic Analysis of Photographed Faces: An Analytical Review

Journal Name: International Journal of Legal Medicine

Author Names: Sean S. Healy & Carl N. Stephan

Affiliation: Laboratory for Human Craniofacial and Skeletal Identification (HuCS-ID Lab), School of Biomedical Sciences, The University of Queensland, Brisbane, Australia, 4072.

Corresponding Author Email: sean.healy@uq.net.au
