# Supplementary material for: Mean human corneal diameter and palpebral fissure lengths as scales for forensic analysis of photographed faces: an analytical review*
Source: Int J Legal Med. 2026 Feb 23;140(3):1529–46. doi: 10.1007/s00414-026-03733-0 (PMC13161299; doi:10.1007/s00414-026-03733-0)
Supplement: Supplementary file 5 — Supplementary Material 5 [file 414_2026_3733_MOESM5_ESM.pdf]

Instrument 1	Instrument 2	Instrument 1 mean (mm)	Instrument 2 mean (mm)	Difference (mm)	Absolute Difference (mm)	Instrument 1 SD (mm)	Instrument 2 SD (mm)	# of studies	# of eyes
IOLMaster 700	Anterion SS-OCT	11.96	11.79	0.18	0.18	0.43	0.45	10	1,175
IOLMaster 700	Pentacam AXL	12.09	11.83	0.26	0.26	0.38	0.42	7	500
IOLMaster 700	Nidek OPD-Scan III	11.98	11.80	0.18	0.18	0.41	0.69	3	1,894
IOLMaster 700	Pentacam	12.00	11.61	0.39	0.39	0.44	0.42	3	1,961
IOLMaster 700	Pentacam HR	11.81	11.45	0.35	0.35	0.44	0.45	3	292
IOLMaster 700	Orbscan II	12.06	11.65	0.42	0.42	0.35	0.32	3	202
IOLMaster 700	Argos SS-OCT	12.14	12.22	-0.08	0.08	0.39	0.53	3	257
IOLMaster 700	Lenstar LS900	11.94	11.91	0.03	0.03	0.49	0.61	2	235
IOLMaster 700	Nidek AL-Scan	12.11	11.98	0.13	0.13	0.37	0.45	2	202
IOLMaster 700	Tomey OA-2000	12.12	12.04	0.07	0.07	0.38	0.46	2	253
IOLMaster 700	Eyestar 900	12.03	12.05	-0.01	0.01	0.41	0.42	2	458
IOLMaster 700	Topcon Aladdin	12.23	11.90	0.34	0.34	0.36	0.35	2	204
IOLMaster 700	Tomey Casia 2 AS-OCT	11.70	11.12	0.58	0.58	0.40	0.65	1	212
IOLMaster 700	Schwind Sirius	12.11	12.01	0.10	0.10	0.43	0.41	1	100
IOLMaster 700	Colombo IOL 2	12.23	11.99	0.24	0.24	0.40	0.35	1	159
IOLMaster 700	Alcon Verion	11.97	11.91	0.06	0.06	0.54	0.49	1	116
IOLMaster 700	Cirrus HD-OCT	12.05	11.88	0.17	0.17	0.30	0.65	1	42
IOLMaster 700	MR-6000	11.70	11.46	0.24	0.24	0.35	0.32	1	30
IOLMaster 700	Cassini Colour LED	11.96	12.65	-0.69	0.69	0.41	0.52	1	49
IOLMaster 700	Visante OCT	12.00	11.79	0.21	0.21	0.30	0.38	1	55
IOLMaster 700	Catalys femtosecond SD-OCT	11.91	11.54	0.37	0.37	0.43	0.49	1	102
IOLMaster 700	IOLMaster 500	12.13	12.26	-0.13	0.13	0.34	0.35	1	116
Anterion SS-OCT	Galilei G6	11.95	12.11	-0.17	0.17	0.21	0.22	2	189
Anterion SS-OCT	Pentacam HR	11.90	11.84	0.06	0.06	0.45	0.48	2	79
Anterion SS-OCT	Pentacam AXL	11.78	11.71	0.08	0.08	0.43	0.40	2	157
Anterion SS-OCT	IOLMaster 500	11.69	12.00	-0.31	0.31	0.45	0.46	1	152
Anterion SS-OCT	Eyestar 900	11.80	12.00	-0.20	0.20	0.40	0.40	1	113
Anterion SS-OCT	Argos SS-OCT	11.67	11.85	-0.18	0.18	0.40	0.37	1	51
Anterion SS-OCT	Lenstar LS900	11.80	12.10	-0.30	0.30	0.40	0.38	1	45
Anterion SS-OCT	Catalys femtosecond SD-OCT	11.76	11.54	0.22	0.22	0.46	0.49	1	102
Anterion SS-OCT	SCHWIND MS-39	12.09	12.04	0.05	0.05	0.45	0.41	1	54
Anterion SS-OCT	Cassini Colour LED	11.84	12.65	-0.81	0.81	0.41	0.52	1	49
Pentacam HR	Tomey Casia 2 AS-OCT	11.33	11.12	0.21	0.21	0.40	0.65	1	212
Pentacam HR	Schwind Sirius	11.72	12.20	-0.48	0.48	0.45	0.53	1	102
Pentacam HR	BQ900	11.74	11.97	-0.23	0.23	0.47	0.59	1	20
Pentacam HR	Galilei G6	12.00	12.30	-0.30	0.30	0.50	0.50	1	30
Pentacam HR	Cassini Colour LED	11.68	12.65	-0.97	0.97	0.38	0.52	1	49
Pentacam AXL	Galilei G6	11.77	12.12	-0.35	0.35	0.59	0.55	2	189
Pentacam AXL	Argos SS-OCT	11.64	12.43	-0.79	0.79	0.39	0.78	2	196
Pentacam AXL	IOLMaster 500	11.33	11.74	-0.41	0.41	0.35	0.39	2	2,498
Pentacam AXL	Topcon Aladdin	12.22	12.02	0.20	0.20	0.41	0.36	1	54
Pentacam AXL	Orbscan II	11.77	11.56	0.21	0.21	0.40	0.03	1	107
Pentacam AXL	Visante OCT	11.70	11.79	-0.09	0.09	0.30	0.38	1	55
Pentacam AXL	Revo 80	11.82	12.85	-1.03	1.03	0.40	0.61	1	144
Lenstar LS900	Nidek OPD-Scan III	11.62	11.60	0.02	0.02	0.56	0.56	1	305
Lenstar LS900	Nidek ARK-1	12.19	12.01	0.18	0.18	0.42	0.44	1	65
Lenstar LS900	Topcon Aladdin	12.27	11.85	0.42	0.42	0.38	0.33	1	150
Lenstar LS900	Nidek AL-Scan	12.27	12.12	0.15	0.15	0.38	0.38	1	150
Lenstar LS900	Argos SS-OCT	12.27	12.14	0.13	0.13	0.38	0.39	1	150
Lenstar LS900	Tomey OA-2000	12.27	12.16	0.11	0.11	0.38	0.36	1	150
Lenstar LS900	Schwind Sirius	11.93	11.72	0.21	0.21	0.39	0.58	1	40
Lenstar LS900	Galilei G4	12.02	12.15	-0.13	0.13	0.43	0.40	1	142
Lenstar LS900	Topcon MYAH	12.28	12.15	0.13	0.13	0.36	0.36	1	40
Topcon Aladdin	Argos SS-OCT	11.74	12.15	-0.41	0.41	0.37	0.45	2	246
Topcon Aladdin	Nidek AL-Scan	11.85	12.12	-0.27	0.27	0.33	0.38	1	150
Topcon Aladdin	Tomey OA-2000	11.85	12.16	-0.31	0.31	0.33	0.36	1	150
IOLMaster 500	Tomey OA-2000	11.46	11.44	0.03	0.03	0.42	0.44	1	90
IOLMaster 500	Galilei G6	12.32	12.21	0.11	0.11	0.31	0.28	1	205
Tomey OA-2000	Suoer SW-9000	11.79	11.58	0.21	0.21	0.38	0.38	1	60
Tomey OA-2000	Nidek AL-Scan	12.16	12.12	0.04	0.04	0.36	0.38	1	150
Tomey OA-2000	Argos SS-OCT	12.16	12.14	0.02	0.02	0.36	0.39	1	150
Schwind Sirius	Scansys Anterior Segment 3D Analyzer	12.17	12.06	0.11	0.11	0.37	0.35	1	38
Schwind Sirius	Orbscan II	12.10	11.76	0.34	0.34	0.45	0.41	1	33
Schwind Sirius	Nidek OPD-Scan III	12.10	12.14	-0.04	0.04	0.45	0.41	1	33
Schwind Sirius	DRI-OCT Triton	12.10	12.40	-0.30	0.30	0.45	0.48	1	33
Schwind Sirius	EyeStar900	12.09	12.23	-0.14	0.14	0.39	0.44	1	74
Schwind Sirius	Schwind MS-39	12.09	12.15	-0.06	0.06	0.39	0.37	1	74
Argos SS-OCT	Eyestar 900	12.75	12.37	0.38	0.38	0.57	0.39	1	56
Argos SS-OCT	Nidek AL-Scan	12.14	12.12	0.02	0.02	0.39	0.38	1	150
Orbscan II	Nidek OPD Scan III	11.79	12.05	-0.26	0.26	0.40	0.41	2	98
Orbscan II	DRI-OCT Triton	11.76	12.40	-0.64	0.64	0.41	0.48	1	33
Orbscan II	MR-6000	11.62	11.46	0.16	0.16	0.53	0.32	1	30
Schwind MS-39	Suoer SW-9000	11.45	11.65	-0.20	0.20	0.37	0.39	1	66
Schwind MS-39	Eyestar 900	12.15	12.23	-0.08	0.08	0.37	0.44	1	74
Nidek OPD Scan III	Pentacam	11.82	11.59	0.23	0.23	0.70	0.38	1	1744
Nidek OPD Scan III	DRI-OCT Triton	12.14	12.40	-0.26	0.26	0.41	0.48	1	11
Nidek OPD Scan III	Galilei G4	12.02	12.15	-0.13	0.13	0.42	0.40	1	142

Title: Mean Human Corneal Diameter and Palpebral Fissure Lengths as Scales for Forensic Analysis of Photographed Faces: An Analytical Review
Journal Name: International Journal of Legal Medicine
Author Names: Sean S. Healy & Carl N. Stephan
Affiliation: Laboratory for Human Craniofacial and Skeletal Identification (HuCS-ID Lab), School of Biomedical Sciences, The University of Queensland, Brisbane, Australia, 4072.
Corresponding Author Email: sean.healy@uq.net.au
