# Supplementary material for: Mean human corneal diameter and palpebral fissure lengths as scales for forensic analysis of photographed faces: an analytical review*
Source: Int J Legal Med. 2026 Feb 23;140(3):1529–46. doi: 10.1007/s00414-026-03733-0 (PMC13161299; doi:10.1007/s00414-026-03733-0)
Supplement: Supplementary file 6 — Supplementary Material 6 [file 414_2026_3733_MOESM6_ESM.docx]

**Supplementary Material 6**

**Palpebral Fissure Anatomy in Brief**

The palpebral fissure is the space between the upper and lower eyelids, which lies anterior to the eye, and provides a view port, for the eye, to the world beyond [1]. The medial and lateral extents of the palpebral fissure (its corners) are defined by the junction sites of the upper and lower eyelids and are respectively termed the medial and lateral canthi. The precise point marking the corner angle at the medial and lateral extents of the palpebral fissure are termed the endo- and exo- (or ecto-) canthions, respectively [2]. The anterior lamella that form the superior and inferior boundaries of the fissure are composed of skin and orbicularis oculi muscle [3]. The posterior lamella, which is positioned deep to the anterior lamella, but still anterior to the eye, contains the tarsal plates that offer dense connective tissue support to the delicate, thin skin of the highly mobile eyelids [4]. Within the posterior lamella is also the conjunctiva and the canthal ligaments, the latter of which helps to suspend and anchor the tarsal plates in place, thereby helping to form the palpebral fissure [3] (Fig. 1).


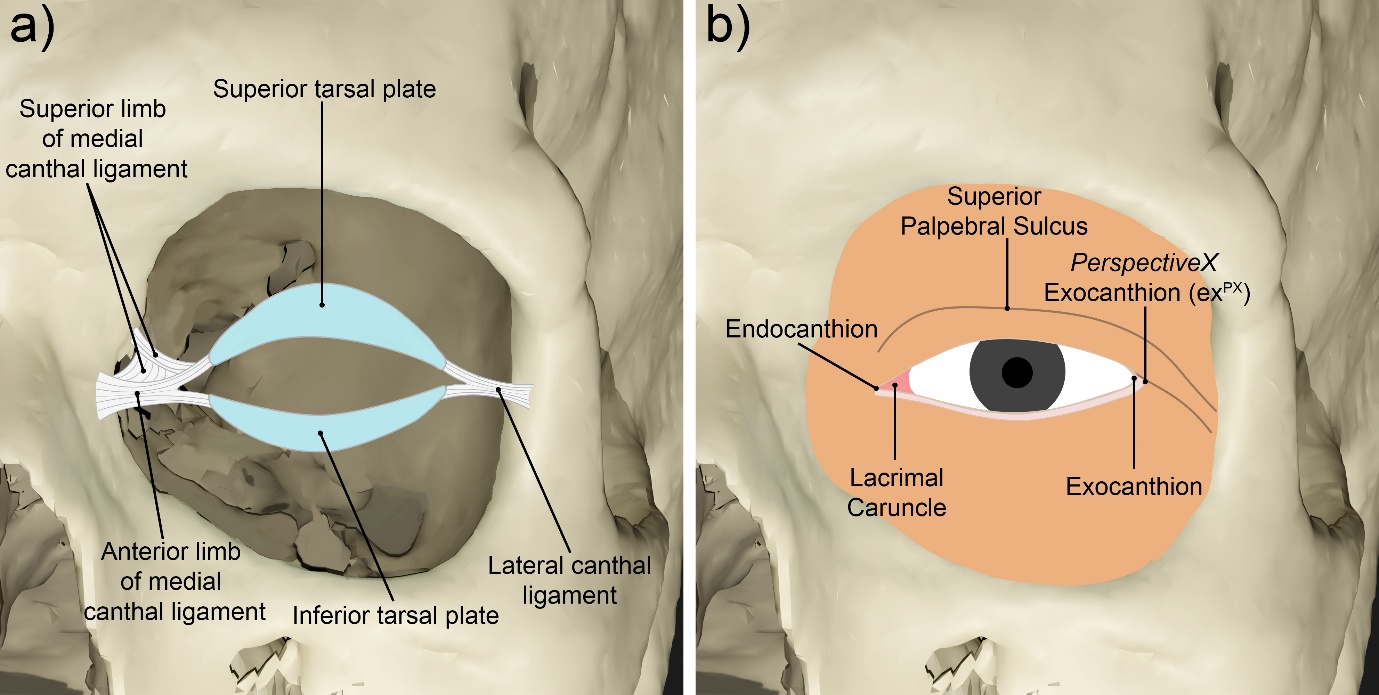
 **Figure 1**. Diagrammatic anatomy of the eyelid’s deep supporting structures and their attachment to the skull: **a** the canthal ligaments and the tarsal plates of the posterior lamella; and **b** the more superficial skin lined eyelids, palpebral fissure and corresponding endo- and exocanthion landmarks.

The canthal ligaments are both Y-shaped in their appearance, although the medial canthal ligament holds a much broader anchoring to the skull than its lateral counterpart. The medial canthal ligament attaches to the skull along the lacrimal crest [5] approximately 10 mm below the dacryon landmark [6], while the lateral canthal ligament anchors the lateral canthus to the malar tubercle (when present) or alternatively at the lateral orbital wall approximately 10 mm below the frontozygomatic suture [3, 4, 6].

The vertical position of the exocanthion (ex) is slightly higher than the endocanthion (en) (mean = 1 mm [7]), but note that this value may be several millimeters larger in some individuals [8-11]. The attachment of the canthal ligaments at their midpoints are at the same vertical height as the canthi, such that the ligaments run horizontally to the orbit [7]. While there are no metric data in the literature on antero-posterior positions of the medial canthal ligament attachments relative to the lateral attachments, adult human skulls clearly exhibit a more forward position of the medial orbital margin, unambiguously flagging that the lateral attachment is more posterior. This is additionally supported, by observations in living subjects via exophthalmometry, that the most anterior point of the corneal apex (oculus anterius) falls 16.2 mm anterior to the deepest point on the lateral orbital margin [12-18]. This relationship awards the palpebral fissure complexity as it wraps in three dimensions laterally around the globe in both posterior and superior trajectories before reaching its termination at the exocanthion. Note here that the palpebral fissure is not a simple vesica piscis shaped opening that fall flatly on a coronal plane (Fig. 2), although it is sometimes measured that way in 2D frontal face photographs (see method 4, 1dPFW, Table 2).


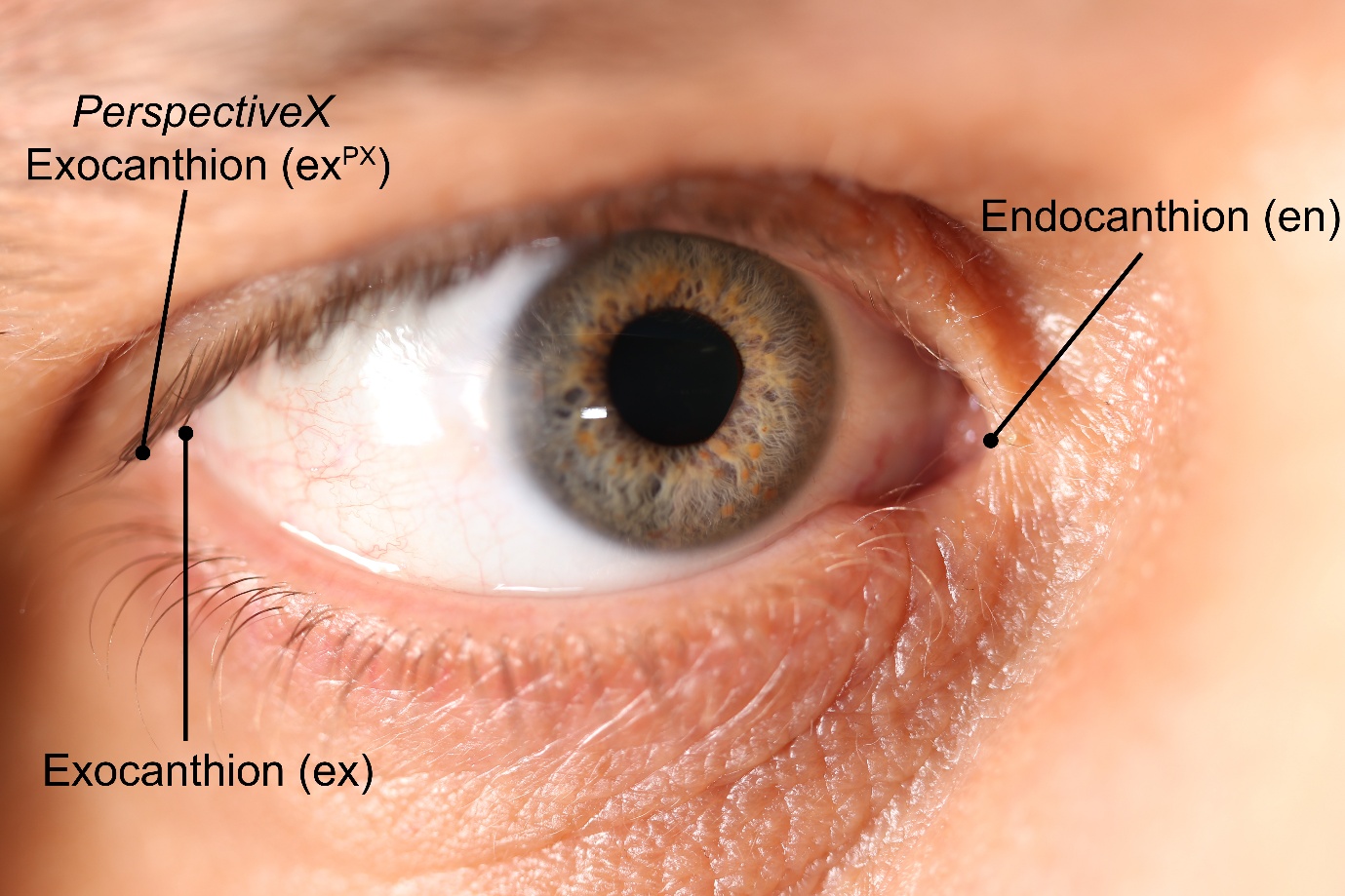


**Figure 2.** Oblique view photograph of eye showing the endo- and exocanthions together with the superior, posterior and lateral wrapping of the lower lid around the eyeball to its junction with the upper eye lid. The anatomical exocanthion is labelled ex. The exocanthion variation used by *PerspectiveX* is labeled ex^PX^.

**References**

1. Rigoni C. (2016) Introduction: Anatomy of the Lips and Eye. In: Fabbrocini G, De Padova MP, Tosti A, eds. Nonsurgical Lip and Eye Rejuvenation Techniques. Springer International Publishing Cham. pp. 1-6.

2. Caple J, Stephan CN (2016) A standardized nomenclature for craniofacial and facial anthropometry. Int J Legal Med 130:863-79. https://doi.org/10.1007/s00414-015-1292-1

3. Cury MC, Sebastiá R. (2021) Anatomy of the Upper and Lower Lids. In: Avelar JM, ed. Aesthetic Facial Surgery. Springer International Publishing Cham. pp. 379-86.

4. Ansari MW, Nadeem A. (2016) Atlas of Ocular Anatomy. Springer International Publishing AG Switzerland. pp. 53-63.

5. George RM (1987) The lateral craniographic method of facial reconstruction. J Forensic Sci 32:1305-30. https://doi.org/10.1520/jfs11181j

6. Stewart TD (1983) The points of attachment of the palpebral ligaments: Their use in facial reconstructions on the skull. J Forensic Sci 28:858-63. https://doi.org/10.1520/jfs11592j

7. Stephan CN, Davidson PL (2008) The placement of the human eyeball and canthi in craniofacial identification. J Forensic Sci 53:612-9. https://doi.org/10.1111/j.1556-4029.2008.00718.x

8. Whitnall SE. (1932) The Anatomy of the Human Orbit and Accessory Organs of Vision. 2nd ed. Oxford University Press London.

9. Wolff E. (1976) Anatomy of the Eye and Orbit. H. K. Lewis & Co London.

10. van den Bosch WA, Leenders I, Mulder P (1999) Topographic anatomy of the eyelids, and the effects of sex and age. Br J Ophthalmol 83:347-52. https://doi.org/10.1136/bjo.83.3.347

11. Pyatkin AN, Dukov DV, Averchenko IV, Veselovska EV (2025) The study of craniofacial correspondence of the eye region structures on anatomical material. Methodological aspects. Herald of Anthropology (Vestnik Antro-pologii) 2:393-403. https://doi.org/10.33876/2311-0546/2025-2/393-403

12. Stephan CN (2002) Facial Approximation: Globe Projection Guideline Falsified by Exophthalmometry Literature. J Forensic Sci 47:1-6. https://doi.org/10.1520/JFS15457J

13. Goldberg RA, Belan A, Hoenig J (1999) Relationship of the eye to the bony orbit, with clinical correlations. Aust N Z J Ophthalmol 27:398-403. https://doi.org/10.1046/j.1440-1606.1999.00243.x

14. Barretto RL, Mathog RH (1999) Orbital measurement in Black and White populations. Laryngoscope 109:1051-4. https://doi.org/10.1097/00005537-199907000-00007

15. Fledelius HC, Stubgaard M (1986) Changes in eye position during growth and adult life as base on exopthalmometry, interpupillary distance, and orbital distance measurements. Acta Opthalmol 64:481-6. https://doi.org/10.1111/j.1755-3768.1986.tb06958.x

16. Migliori ME, Gladstone GJ (1984) Determination of the normal range of exophthalmometric values for Black and White adults. Am J Ophthalmol 988:438-42. https://doi.org/10.1016/0002-9394(84)90127-2

17. Brown RD, Douglas J (1975) Exophthalmometry of Blacks. Ann Intern Med 83:835-6. https://doi.org/10.7326/0003-4819-83-6-835_2

18. Knudtzon K (1949) On exophthalmometry: the result of 724 measurements with Hertel's exophthalmometer On normal adult individuals. Acta Psychiatr Scand 24:523-37. https://doi.org/10.1111/j.1600-0447.1949.tb07336.x

Title: Mean Human Corneal Diameter and Palpebral Fissure Lengths as Scales for Forensic Analysis of Photographed Faces: An Analytical Review

Journal Name: International Journal of Legal Medicine

Author Names: Sean S. Healy & Carl N. Stephan

Affiliation: Laboratory for Human Craniofacial and Skeletal Identification (HuCS-ID Lab), School of Biomedical Sciences, The University of Queensland, Brisbane, Australia, 4072.

Corresponding Author Email: sean.healy@uq.net.au
