# Supplementary material for: Mean human corneal diameter and palpebral fissure lengths as scales for forensic analysis of photographed faces: an analytical review*
Source: Int J Legal Med. 2026 Feb 23;140(3):1529–46. doi: 10.1007/s00414-026-03733-0 (PMC13161299; doi:10.1007/s00414-026-03733-0)
Supplement: Supplementary file 7 — Supplementary Material 7 [file 414_2026_3733_MOESM7_ESM.docx]

**Supplementary Material 7**

**Mean Adult Palpebral Fissure dimensions statistics with References**

**Table 1:** Mean dimensions for each one of the four palpebral fissure measurement classes (see Table 2) in adults.

| **Measurement** | **Group** | **Weighted mean (mm)** | **Combined SD (mm)** | **Number of eyes (n)** | **Number of studies (n)** | **Studies** |
| --- | --- | --- | --- | --- | --- | --- |
| 3dPFL | M | 33.0 | 2.9 | 1,239 | 5 | [1-5] |
|  | F | 32.0 | 2.3 | 1,117 | 5 | [1-5] |
|  | Combined Sexes (M+F) | 32.5 | 2.7 | 2,356 | 5 | [1-5] |
| 2dPFW(a) | M | 30.2 | 3.8 | 2,015 | 8 | [6-13] |
|  | F | 28.9 | 3.6 | 2,277 | 9 | [6-14] |
|  | Combined Sexes (M+F) | 29.5 | 3.7 | 4,548 | 11 | [6-16] |
| 2dPFW(b) | M | 29.2 | 2.7 | 1,641 | 9 | [17-25] |
|  | F | 28.1 | 2.8 | 2,387 | 10 | [17-26] |
|  | Combined Sexes (M+F) | 27.4 | 3.2 | 5,871 | 14 | [17-30] |
| 1dPFW | M | 28.6 | 2.7 | 463 | 6 | [31-36] |
|  | F | 27.2 | 3.8 | 751 | 6 | [31-36] |
|  | Combined Sexes (M+F) | 27.7 | 3.3 | 1,354 | 8 | [31-37] |

**References**

1. Sforza C, Dolci C, Grandi G, Tartaglia GM, Laino A, Ferrario VF (2015) Comparison of soft-tissue orbital morphometry in attractive and normal Italian subjects. Angle Orthod 85:127-33. https://doi.org/10.2319/012814-75.1

2. Ferrario VF, Sforza C, Colombo A, Schmitz JH, Serrao G (2001) Morphometry of the orbital region: A soft-tissue study from adolescence to mid-adulthood. Plast Reconstr Surg 108:285-92. https://doi.org/10.1097/00006534-200108000-00001

3. Sforza C, Grandi G, Catti F, Tommasi DG, Ugolini A, Ferrario VF (2009) Age- and sex-related changes in the soft tissues of the orbital region. Forensic Sci Int 185:115.e1-.e8. https://doi.org/10.1016/j.forsciint.2008.12.010

4. Purkait R (2013) Growth Pattern of the Eye from Birth to Maturity: An Indian Study. Asthetic Plast Surg 37:128-34. https://doi.org/10.1007/s00266-012-0010-3

5. Farkas LG, Hreczko TM, Katic M. (1994) Craniofacial norms in North American Caucasians from birth (one year) to young adulthood. In: Farkas LG, ed. Anthropometry of the Head and Face. Raven Press New York. pp. 241-336.

6. Bozkir MG, Karakaş P, Oĝuz Ö (2003) Measurements of soft tissue orbits in Turkish young adults. Surg Radiol Anat 25:54-7. https://doi.org/10.1007/s00276-002-0092-8

7. Jiang A, Zhang F, Kurbana M, Xiong K (2023) Normal palpebral anthropometric measurements in Uygur population: A cross-sectional study. J Pak Med Assoc 73:796-9. https://doi.org/10.47391/JPMA.6185

8. Barretto RL, Mathog RH (1999) Orbital measurement in Black and White populations. Laryngoscope 109:1051-4. https://doi.org/10.1097/00005537-199907000-00007

9. Gudek MA, Uzun A (2015) Anthropometric measurements of the orbital contour and canthal distance in young Turkish. J Anat Soc India 64:S1-S6. https://doi.org/10.1016/j.jasi.2014.09.007

10. Al-Sebaei MO (2015) The validity of three neo-classical facial canons in young adults originating from the Arabian Peninsula. Head Face Med 11:4. https://doi.org/10.1186/s13005-015-0064-y

11. Bozkir MG, Karakas P, Oguz Ö (2004) Vertical and horizontal neoclassical facial canons in Turkish young adults. Surg Radiol Anat 26:212-9. https://doi.org/10.1007/s00276-003-0202-2

12. Farkas LG, Lindsay WK, Vanderby MB (1972) Morphology of the orbital region in adults following the cleft lip/palate repair in childhood. Am J Phys Anthropol 37:65-73. https://doi.org/10.1002/ajpa.1330370109

13. Lu D-W, Shi B, Chen H-Q, He X, Liao L-S, Zheng Q (2010) A comparative study of fluctuating asymmetry in Chinese families with nonsyndromic ceft palate. Cleft Palate Craniofac J 47:182-8. https://doi.org/10.1597/08-197_1

14. Kim YC, Kwon JG, Kim SC, Huh CH, Kim HJ, Oh TS, Koh KS, Choi JW, Jeong WS (2018) Comparison of periorbital anthropometry between beauty pageant contestants and ordinary young women with korean ethnicity: A three-dimensional photogrammetric analysis. Aesthetic Plast Surg 42:479-90. https://doi.org/10.1007/s00266-017-1040-7

15. Le TT, Farkas LG, Ngim RCK, Levin LS, Forrest CR (2002) Proportionality in Asian and North American Caucasian faces using neoclassical facial canons as criteria. Aesthetic Plast Surg 26:64-9. https://doi.org/10.1007/s00266-001-0033-7

16. Shaner DJ, Peterson AE, Beattie OB, Bamforth JS (1998) Facial measurements in clinical genetics: How important are the instruments we use? Am J Med Genet 77:384-90. https://doi.org/10.1002/(SICI)1096-8628(19980605)77:5<384::AID-AJMG6>3.0.CO;2-N

17. Knezi N, Isaretovic V, Adjic I, Babic N, Maric D, Stojsic-Dzunja L (2020) Morphometric analysis of the palpebral fissure and canthal distance in Serbian young adults. International Journal of Morphology 38:1381-5. https://doi.org/10.4067/S0717-95022020000501381.

18. Vasanthakumar P, Kumar P, Rao M (2013) Anthropometric analysis of palpebral fissure dimensions and its position in South Indian ethnic adults. Oman Med J 28:26-32. https://doi.org/10.5001/omj.2013.06

19. Wu X-S, Jian X-C, He Z-J, Gao X, Li Y, Zhong X (2010) Investigation of anthropometric measurements of anatomic structures of orbital soft tissue in 102 young Han Chinese adults. Ophthalmic Plast Reconstr Surg 26:339-43. https://doi.org/10.1097/IOP.0b013e3181c94e97

20. Price KM, Gupta PK, Woodward JA, Stinnett SS, Murchison AP (2009) Eyebrow and eyelid dimensions: an anthropometric analysis of African Americans and Caucasians. Plast Reconstr Surg 124:615-23. https://doi.org/10.1097/PRS.0b013e3181addc98

21. Song W, Kim S, Kim S, Hu K, Kim H, Koh K (2007) Asymmetry of the palpebral fissure and upper eyelid crease in Koreans. J Plast Reconstr Aesthet Surg 60:251-5. https://doi.org/10.1016/j.bjps.2006.04.027

22. Kunjur J, Sabesan T, Ilankovan V (2006) Anthropometric analysis of eyebrows and eyelids: An inter-racial study. Br J Oral Maxillofac Surg 44:89-93. https://doi.org/10.1016/j.bjoms.2005.03.020

23. Packiriswamy V, Kumar P, Rao KM (2012) Photogrammetric analysis of palpebral fissure dimensions and its position in Malaysian South Indian ethnic adults by gender. N Am J Med Sci 4:458. https://doi.org/10.4103/1947-2714.101984

24. Packiriswamy V, Kumar P, Bashour M (2018) Anthropometric and anthroposcopic analysis of periorbital features in Malaysian population: An inter-racial study. Facial Plast Surg 34:400-6. https://doi.org/10.1055/s-0038-1648224

25. Kuǧu N, Sonmez M, Erdogan H (2004) Measurements of the orbital soft tissue in schizophrenic patients. Neurology, Psychiatry, and Brain Research 11:199-204. https://doi.org/''

26. Direk FK, Deniz M, Uslu AI, Doğru S (2016) Anthropometric analysis of orbital region and age-related changes in adult women. J Craniofac Surg 27:1579-82. https://doi.org/10.1097/SCS.0000000000002814

27. Yu P, Nathan P, Meng CS (2019) Periocular anthropometry of normal Chinese and Indian populations in Singapore. JOJ Ophthalmol 7:555722. https://doi.org/10.19080/JOJO.2019.07.555722

28. Rana K, Beecher M, Caltabiano C, Macri C, Zhao Y, Verjans J, Selva D (2024) Artificial intelligence to automate assessment of ocular and periocular measurements. Eur J Ophthalmol 35:346-51. https://doi.org/10.1177/11206721241249773

29. Viveiros MMH, Matai O, Takahagi RU, Padovani CR, Schellini SA (2017) Eyelid fissure dimensions in Japanese and in Brazilians of European descent over 50 years of age. Arq Bras Oftalmol 80:304-8. https://doi.org/10.5935/0004-2749.20170074

30. DeAngelis KD, Rider A, Potter W, Jensen J, Fowler BT, Fleming JC (2019) Eyelid spontaneous blink analysis and age-related changes through high-speed imaging. Ophthalmic Plast Reconstr Surg 35:487-90. https://doi.org/10.1097/IOP.0000000000001349

31. Ozdemir T, Can FE, Isiklar S, Ercan I, Cankur NS (2017) Periorbital soft tissue anthropometric analysis of young adults. J Craniofac Surg 28:e311-e8. https://doi.org/10.1097/SCS.0000000000003558

32. Ozdemir F, Golpinar M, Nahir M, Sahin B (2022) Anthropometric Periocular Soft Tissue Analysis From Preadolescence to Young Adulthood: Photogrammetric Measurements. J Craniofac Surg 33:2045-8. https://doi.org/10.1097/SCS.0000000000008504

33. Tran C, Choi D, Wang K, Carter KD, Ko AC, Shriver EM (2023) Trends in horizontal periocular asymmetry. Can J Ophthalmol 58:229-34. https://doi.org/10.1016/j.jcjo.2021.11.004

34. Erbagci I, Erbagci H, Kizilkan N, Gumusburun E, Bekir N (2005) The effect of age and gender on the anatomic structure of Caucasian healthy eyelids. Saudi Med J 26:1535-8. https://doi.org/''

35. Ing E, Safarpour A, Ing T, Ing S (2006) Ocular adnexal asymmetry in models: a magazine photograph analysis. Can J Ophthalmol 41:175-82. https://doi.org/10.1139/I06-005

36. Li Q, Zhang X, Li K, Quan Y, Cai X, Xu S, Zhu F, Lu R (2016) Normative anthropometric analysis and aesthetic indication of the ocular region for young Chinese adults. Graefes Arch Clin Exp Ophthalmol 254:189-97. https://doi.org/10.1007/s00417-015-3179-8

37. Read SA, Collins MJ, Carney LG (2006) The morphology of the palpebral fissure in different directions of vertical gaze. Optom Vis Sci 83:715-22. https://doi.org/10.1097/01.opx.0000236811.78177.97

Title: Mean Human Corneal Diameter and Palpebral Fissure Lengths as Scales for Forensic Analysis of Photographed Faces: An Analytical Review

Journal Name: International Journal of Legal Medicine

Author Names: Sean S. Healy & Carl N. Stephan

Affiliation: Laboratory for Human Craniofacial and Skeletal Identification (HuCS-ID Lab), School of Biomedical Sciences, The University of Queensland, Brisbane, Australia, 4072.

Corresponding Author Email: sean.healy@uq.net.au
