# Supplementary material for: Mean human corneal diameter and palpebral fissure lengths as scales for forensic analysis of photographed faces: an analytical review*
Source: Int J Legal Med. 2026 Feb 23;140(3):1529–46. doi: 10.1007/s00414-026-03733-0 (PMC13161299; doi:10.1007/s00414-026-03733-0)
Supplement: Supplementary file 8 — Supplementary Material 8 [file 414_2026_3733_MOESM8_ESM.docx]

**Supplementary Material 8**

**Mean Subadult Palpebral Fissure Dimensions Statistics with References**

**Table 1:** Mean dimensions for each one of the four palpebral fissure measurement classes (see Table 2) in subadults.

| **Measurement** | **Weighted mean (mm)** | **Combined SD (mm)** | **Number of eyes (n)** | **Number of studies (n)** | **Studies** |
| --- | --- | --- | --- | --- | --- |
| 3dPFL | 29.4 | 3.1 | 4258 | 4 | [1-4] |
| 2dPFW(a) | 25.9 | 1.0 | 3510 | 3 | [5-7] |
| 2dPFW(b) | 28.6 | 3.3 | 1844 | 1 | [8] |
| 1dPFW | 26.9 | 2.7 | 524 | 3 | [9-11] |

**References**

1. Blanck-Lubarsch M, Dirksen D, Feldmann R, Sauerland C, Hohoff A (2019) 3D-Analysis of mouth, nose and eye parameters in children with fetal alcohol syndrome (FAS). Int J Environ Res Public Health 16:2535. https://doi.org/10.3390/ijerph16142535

2. Sforza C, Grandi G, Catti F, Tommasi DG, Ugolini A, Ferrario VF (2009) Age- and sex-related changes in the soft tissues of the orbital region. Forensic Sci Int 185:115.e1-.e8. https://doi.org/10.1016/j.forsciint.2008.12.010

3. Ferrario VF, Sforza C, Colombo A, Schmitz JH, Serrao G (2001) Morphometry of the orbital region: A soft-tissue study from adolescence to mid-adulthood. Plast Reconstr Surg 108:285-92. https://doi.org/10.1097/00006534-200108000-00001

4. Farkas LG, Hreczko TM, Katic M. (1994) Craniofacial norms in North American Caucasians from birth (one year) to young adulthood. In: Farkas LG, ed. Anthropometry of the Head and Face. Raven Press New York. pp. 241-336.

5. Abou El Ella SS, Tawfik MA, El Shaheed MYA, Barseem NF (2022) Validated establishment of anthropometric measurements of mid-face zone in Egyptian healthy preschool-age children: A cross-sectional study. Egypt J Med Hum Genet 23:80. https://doi.org/10.1186/s43042-022-00294-w

6. Raffa LH, Hellström A, Aring E, Andersson S, Andersson Grönlund M (2014) Ocular dimensions in relation to auxological data in a sample of Swedish children aged 4–15 years. Acta Ophthalmol 92:682-8. https://doi.org/10.1111/aos.12310

7. Andersson Grönlund M, Landgren M, Strömland K, Aring E, Svensson L, Tuvemo T, Hellström A (2010) Relationships between ophthalmological and neuropaediatric findings in children adopted from Eastern Europe. Acta Ophthalmol 88:227-34. https://doi.org/10.1111/j.1755-3768.2008.01430.x

8. Purkait R (2013) Growth Pattern of the Eye from Birth to Maturity: An Indian Study. Asthetic Plast Surg 37:128-34. https://doi.org/10.1007/s00266-012-0010-3

9. Li H, Shi S, Lou L, Cao J, Zhou Z, Huang X, Ye J (2024) Multidimensional quantitative characterization of periocular morphology: distinguishing esotropia from epicanthus by deep learning network. Quant Imaging Med Surg 14:6273-84. https://doi.org/10.21037/qims-24-155

10. Ozdemir F, Golpinar M, Nahir M, Sahin B (2022) Anthropometric Periocular Soft Tissue Analysis From Preadolescence to Young Adulthood: Photogrammetric Measurements. J Craniofac Surg 33:2045-8. https://doi.org/10.1097/SCS.0000000000008504

11. Erbagci I, Erbagci H, Kizilkan N, Gumusburun E, Bekir N (2005) The effect of age and gender on the anatomic structure of Caucasian healthy eyelids. Saudi Med J 26:1535-8. https://doi.org/''

Title: Mean Human Corneal Diameter and Palpebral Fissure Lengths as Scales for Forensic Analysis of Photographed Faces: An Analytical Review

Journal Name: International Journal of Legal Medicine

Author Names: Sean S. Healy & Carl N. Stephan

Affiliation: Laboratory for Human Craniofacial and Skeletal Identification (HuCS-ID Lab), School of Biomedical Sciences, The University of Queensland, Brisbane, Australia, 4072.

Corresponding Author Email: sean.healy@uq.net.au
